# Non‐Abrupt Vegetation Changes due to Altered Nutrient Balance Make Complex Scale‐Dependent Warming and Cooling Effects

**DOI:** 10.1111/gcb.70782

**Published:** 2026-03-17

**Authors:** Bayu Hanggara, Tarek El‐Madany, Arnaud Carrara, Gerardo Moreno, Rosario Gonzalez‐Cascon, Vicente Burchard‐Levine, M. Pilar Martin, Stefan Metzger, Anke Hildebrandt, Markus Reichstein, Sung‐Ching Lee

**Affiliations:** ^1^ Department of Biogeochemical Integration Max Planck Institute for Biogeochemistry Jena Germany; ^2^ Faculty of Chemistry and Earth Science Friedrich‐Schiller University Jena Germany; ^3^ Fundacion Centro de Estudios Ambientales del Mediterráneo (CEAM) Valencia Spain; ^4^ Faculty of Forestry, Institute of Dehesa Research (INDEHESA) Universidad de Extremadura Plasencia Spain; ^5^ National Institute for Agriculture and Food Research and Technology (INIA‐CSIC) Madrid Spain; ^6^ Spanish National Research Council Environmental Remote Sensing and Spectroscopy Laboratory (SpecLab) Madrid Spain; ^7^ Department of Atmospheric and Oceanic Sciences University of Wisconsin–Madison Madison Wisconsin USA; ^8^ Atmofacts Longmont Colorado USA; ^9^ Department Computational Hydrosystems Helmholtz Centre for Environmental Research (UFZ) Leipzig Germany

## Abstract

Land‐atmosphere exchanges are mediated by biophysical properties (e.g., albedo change, evaporative cooling) and biogeochemical cycle (e.g., CO_2_ fluxes), with both processes exerting global feedback as radiative forcing (RF). While most research on RF concentrated on the impact of abrupt vegetation changes, this study investigates the effects on non‐abrupt changes due to altered nutrient levels (i.e., nitrogen [N] and phosphorus [P] deposition). We examined impacts of these changes by assessing RF, representing global effects, and linked it with surface temperature (Ts), reflecting local influence. We hypothesized there are scale‐dependent warming and cooling effects due to surface‐atmosphere interactions. We explored this question using a 9‐year dataset (2014–2023) from a large‐scale nutrient manipulation experiment in a semi‐arid savanna, Spain. Three co‐located eddy‐covariance sites are established: control, N‐added (NT), and N+P‐added (NPT). The results indicate domination of changes in surface albedo over CO_2_ fluxes, producing paradoxical effects: a net cooling at global scale (RF differences are [mean ± SD]—0.46 ± 0.08 W m^−2^ [global] m^−2^ [surface] at NT and −0.39 ± 0.09 W m^−2^ m^−2^ at NPT) due to higher surface reflectivity, but localized warming at understory (Ts differences are 0.63°C ± 0.46°C at NT and 0.80°C ± 0.77°C at NPT) driven by shifts in energy partitioning. Furthermore, our findings indicate that N‐only addition has more canopy‐level Ts cooling than N+P treatment, although Ts increases at the understory. These contrasting responses reveal a layered and scale‐dependent interplay of surface‐atmosphere interactions. They highlight the critical role of nutrient stoichiometry in shaping climate feedbacks despite the vegetation changes are not abrupt, and emphasize that what cools the globe may still warm the land beneath our feet.

## Graphical abstract

Semi‐arid ecosystems are particularly vulnerable to elevated N deposition. However, there is still a critical gap in understanding the impact of N:P imbalance by considering biophysical changes and biogeochemical cycle. This study presents the complex scale‐dependent warming and cooling effects due to altered nutrient balance on the Mediterranean semi‐arid ecosystem. The arrows represent the values being higher or lower at the nutrient addition than the control sites, and asterisk marks indicate the differences are significant across whole seasons. These contrasting responses reveal a layered and scale‐dependent interplay of surface‐atmosphere interactions. They highlight the critical role of nutrient stoichiometry in shaping climate feedback despite the vegetation changes not being abrupt, and emphasize that what cools the globe may still warm the land beneath our feet.

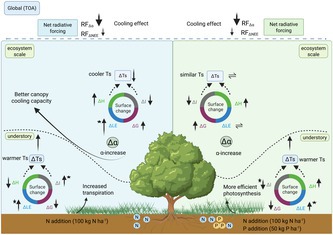

## 1 Introduction

Climate change and human activities have significantly altered the global nitrogen (N) and phosphorus (P) cycle. Those perturbations, in particular enhanced N deposition, lead to N:P imbalances in many terrestrial ecosystems, which can affect the ecosystem structure (Guignard et al. 2017; Penuelas et al. 2020), phenological phases (Luo et al. 2020; Wang and Tang 2019), and photosynthetic capacity (Sun et al. 2022; Yang et al. 2016). Additionally, N:P imbalances also drive the changes in biophysical (e.g., surface albedo [α], evaporative cooling) and biogeochemical processes (e.g., carbon dioxide [CO_2_] fluxes) which can have a substantial impact on land‐atmosphere interactions on multiple scales.

Semi‐arid ecosystems are particularly vulnerable to elevated N deposition (Erisman et al. 2013). These ecosystems also play an important role in the interannual variability of the global carbon (C) cycle (Ahlstrom et al. 2015; Poulter et al. 2014; Sitch et al. 2024). Recent studies have advanced our understanding of the impact of increasing N deposition on biogeochemical cycle, such as CO_2_ fluxes (El‐Madany et al. 2021; Nair et al. 2024), ecosystem water use efficiency (WUE) (Rastetter et al. 2022; Scaini et al. 2023), and biophysical processes (i.e., senescence dynamics [Luo et al. 2020]). However, there is still a critical gap in understanding the consequences of N:P imbalance by considering processes from both biophysical changes and biogeochemical cycles. Therefore, a holistic framework that integrates them is essential to simulate the response of semi‐arid ecosystems to global change.

Biophysical processes are primarily associated with surface‐atmosphere energy (e.g., evaporative cooling and radiative exchange) and momentum (e.g., surface roughness and turbulent fluxes) interactions, while biogeochemical cycles involve the exchanges of greenhouse gases (Luyssaert et al. 2018). Biogeochemical dynamics are often referred to changes in CO_2_ absorption, whereas α is a key biophysical property along with roughness and evapotranspiration (Graf et al. 2023; Kan et al. 2025). Biophysical feedback could enhance, offset, or counteract the biogeochemical impact at the top of atmosphere (TOA) expressed as radiative forcing (RF) (Chen et al. 2020; Forzieri et al. 2017). N:P imbalance could gradually modify surface biophysical properties and biogeochemical cycle; however, our understanding on those effects on climate feedback is still limited. Most studies on terrestrial climate feedback were still focused on abrupt vegetation conversion such as deforestation (Kirschbaum et al. 2011; Scott et al. 2018), afforestation (Wang et al. 2023; Zheng et al. 2020), agriculture system (Lugato et al. 2020; Yu et al. 2024), wind‐throw (Ney et al. 2019), and snow impact (Gnanamoorthy et al. 2021). Considering that the N:P imbalance influences the phenological response of vegetation (Luo et al. 2020), it is essential to assess its impacts on climate feedback to advance our understanding of this non‐abrupt modification of vegetation over an extended period.

RF is conventionally assessed at the TOA, making it suitable for representing large‐scale climate impacts. However, it exhibits limitations in capturing the finer‐scale variations in local conditions (Shindell et al. 2015). More critically, changes in surface temperature (∆Ts) and their patterns may not consistently correspond with the warming or cooling trends indicated at TOA by RF (Campra et al. 2008). ∆Ts can be much more sensitive to changes in partitioning of available energy into sensible (H) and latent heat (LE) than changes in total available energy only (Li and Wang 2019). The biophysical feedbacks are also often highly localized, not only influenced by surface α but also turbulent cooling effect and aerodynamic resistance (Alkama and Cescatti 2016; Chen et al. 2020; Duveiller et al. 2018). The relationships between changes in surface α and net ecosystem exchange (NEE) are often inverse (Graf et al. 2023), but some studies demonstrated a joint cooling effect (Carrer et al. 2018; Genesio et al. 2020). Therefore, it is essential to integrate biophysical and biogeochemical perspectives via net RF and ∆Ts to achieve a comprehensive understanding of the multiscale climatic impacts of N:P imbalance.

**FIGURE 1:**
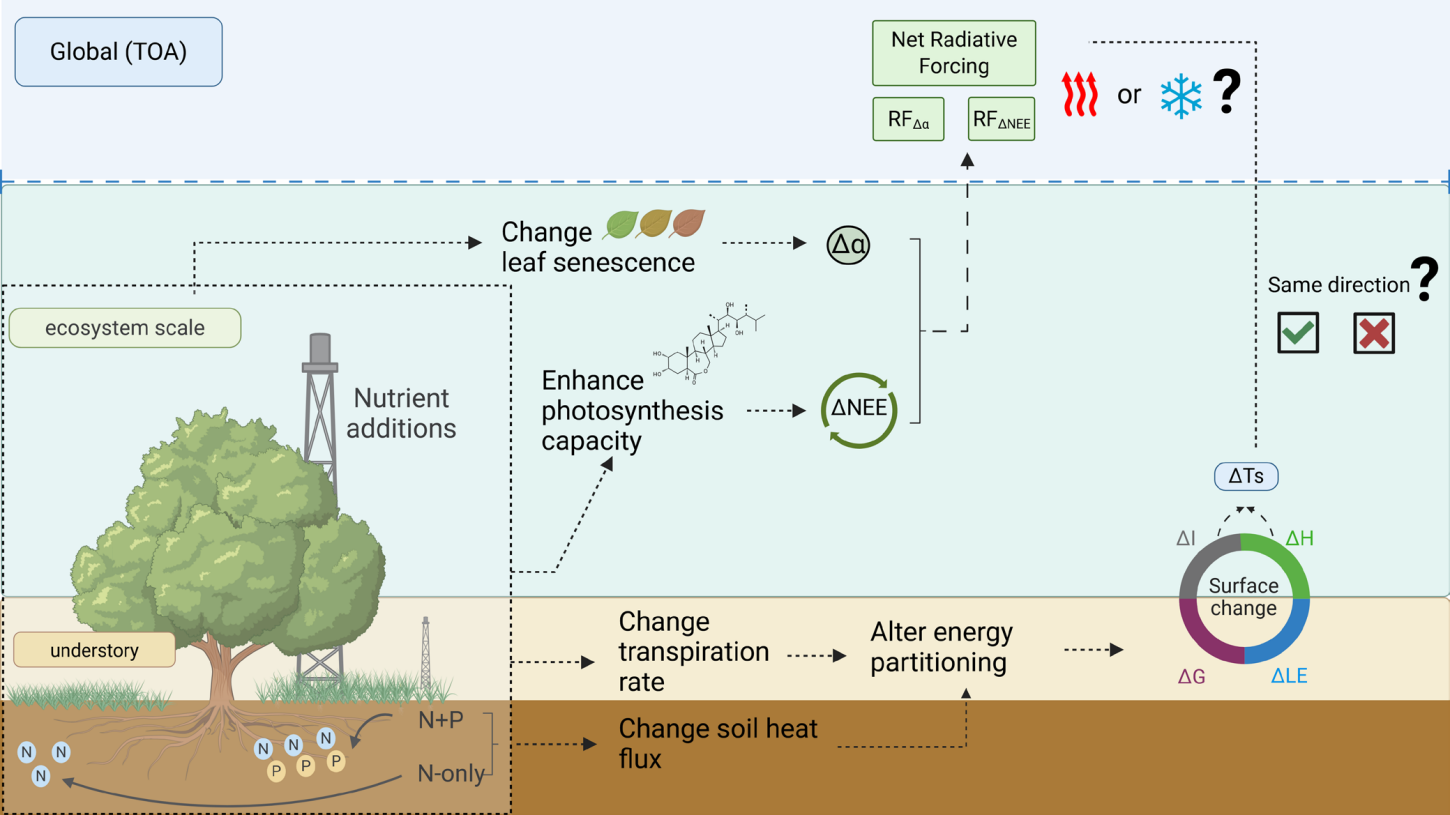
Illustration of hypothesis and research questions in this study. Nutrient addition (i.e., nitrogen [***N***] and phosphorus [***P***]) affects surface‐atmosphere interaction through energy fluxes dynamics such as changes in latent heat (∆LE), sensible heat (∆H), soil heat flux (∆G), residual energy imbalance (∆I), which lead to surface temperature change (∆Ts). It also influences the atmosphere via combination of the impacts caused by surface albedo change (∆α) and net CO_2_ uptake (∆NEE), represented using radiative forcing (RF) at the top of atmosphere (TOA).

To address this knowledge gap, an ecosystem‐scale nutrient manipulation experiment has been established since the end of 2014 in a semi‐arid tree‐grass savanna located in Majadas de Tiétar, South‐West Spain. The experiment comprises three sites: one receiving N‐only addition (i.e., representing atmospheric N deposition and resulting N:P imbalance), another one receiving both N and P addition (i.e., alleviating the imbalance at a higher nutrient level), and a control site without any manipulation (El‐Madany et al. 2018; Luo et al. 2020). Our study investigates how the semi‐arid savanna ecosystem responds to nutrient addition particularly on two integrated layers—ecosystem scale (i.e., tree‐grass coexistence) and understory scale (i.e., grass layer). We analyzed 9‐year (2014–2023) time series of CO_2_ and energy fluxes with biometeorological variables from the three sites, intending to address the following research questions for semi‐arid savannas (Figure 1):

1. What are the impacts of nutrient imbalance on warming or cooling effect via net RF at TOA (i.e., global scale) based on changes from both biophysical (i.e., ∆α) and biogeochemical properties (i.e., ∆NEE)?
2. How does the nutrient imbalance affect ∆Ts of ecosystem and understory scales (i.e., local scale) as it alters the radiative and energy fluxes partitioning?
3. Which is the dominant driver that contributes to ∆Ts and how do the energy flux partitioning differ between the understory (i.e., single layer vegetation) and ecosystem scales (i.e., tree‐grass coexistence)?

We hypothesized due to the nature of semi‐arid savanna as a water‐nutrient limited ecosystem, N:P imbalance can enhance surface reflectivity and CO_2_ absorption, resulting in a cooling effect at TOA (Figure 1). Meanwhile, tree‐grass coexistence creates a different energy flux partitioning between the ecosystem and understory scales. Therefore, our second hypothesis proposed that substantial changes in non‐radiation factors (i.e., energy fluxes) can lead to different net warming or cooling effects (i.e., ∆Ts) across various ecosystem strata.

## 2 Material and Methods

### 2.1 Description of the Experimental Site

The Majadas de Tiétar research site is a typical “*Iberic Dehesa*,” characterized by a combination of a herbaceous layer of native pasture and a tree layer of scattered evergreen oak trees, with 98% of these being *Quercus ilex* . This ecosystem features a tree density of approximately 20–25 trees ha^−1^, with an average diameter at breast height of 46 cm and mean canopy height of around 8.7 m. The trees are generally equally distributed throughout the ecosystem, although some areas may exhibit random clustering or openness. The tree canopy fraction is roughly about 23% (Bogdanovich et al. 2021). Meanwhile, the understory layer consists mostly of various annual native species, dominated by three main functional plant forms: grasses, forbs, and legumes. The understory layer varies both spatially and temporally in accordance to their phenological phases (El‐Madany et al. 2018; Perez‐Priego et al. 2015). The tree leaf area index (LAI) is approximately 0.35 m^2^ m^−2^ (1.5–2.0 m^2^ m^−2^ on a tree level) and the understory layer has LAI values between 0.5 and 2.5 m^2^ m^−2^ with a peak in spring (Migliavacca et al. 2017). This site is considered heterogeneous at spatial scales from centimeters to tens of meters, with large variability in plant species and their distribution within the herbaceous layer (Migliavacca et al. 2017). However, it can be considered homogeneous at a scale of hundreds of meters (El‐Madany et al. 2018) (Table 1).

**TABLE 1:** Nomenclature variables used in this study.

Variable notation	Unit	Definition
Carbon flux partitioning		
*NEE*	g C m^−2^ d^−1^	Net ecosystem exchange
*GPP*	g C m^−2^ d^−1^	Gross primary productivity
Reco	g C m^−2^ d^−1^	Ecosystem respiration
Radiative forcing		
${\mathrm{RF}}_{\Delta \alpha }$	W m^−2^ (global) m^−2^ (surface)	Global effect of radiative forcing at TOA of each square meter of treated land surface due to surface albedo change
${\mathrm{RF}}_{\text{ΔNEE}}$	W m^−2^ (global) m^−2^ (surface)	Global effect of radiative forcing of each square meter of treated land surface due to NEE change
${\mathrm{RF}}_{m}$	W m^−2^ (global)	Global effect of radiative forcing at monthly timescale due to albedo and NEE changes
∑RF	W m^−2^ (global) m^−2^ (surface)	Cumulative global effect of radiative forcing of each square meter of treated land surface. Combination due to albedo and NEE changes
*β*	Unitless	Airborne fraction
$M_{a}$, $M_{c}$		Molar masses of air and carbon
$m_{a}$	5.15 × 10^21^ g	Mass of the atmosphere
$\chi_{0},{\mathrm{CO}}_{2}$	ppm	Base concentration of CO_2_ in the atmosphere
${\mathrm{GWP}}_{\Delta \alpha }$	kg CO_2_ ha^−1^ yr^−1^	Global warming potential due to surface albedo change
Radiometric and energy components		
Rn	W m^−2^	Net radiation
SWUR,LWUR	W m^−2^	Shortwave and longwave outgoing radiation
ΔSWDR,δSWDR	W m^−2^, °C	Difference in incoming shortwave radiation as energy and degree temperature unit, respectively
ΔLWDR,δLWDR	W m^−2^, °C	Difference in incoming longwave radiation as energy and degree temperature unit, respectively
*Δα*, *δα*	Unitless, °C	Difference in surface albedo and in degree temperature unit, respectively
ΔLE, δLE	W m^−2^, °C	Difference in latent heat as energy and in degree temperature unit, respectively
*ΔH*, *δH*	W m^−2^, °C	Difference in sensible heat as energy and in degree temperature unit, respectively
*ΔG*, *δG*	W m^−2^, °C	Difference in soil heat flux as energy and in degree temperature unit, respectively
*ΔI*, *δI*	W m^−2^, °C	Difference in energy imbalance as energy and in degree temperature unit, respectively
*Δ* ε, *δ* ε	W m^−2^, °C	Difference in emissivity as energy and in degree temperature unit, respectively
Surface temperature		
${\mathrm{\Delta T}}_{s,\mathrm{cal}}$	°C	Surface temperature change calculated by incorporating the incoming shortwave radiations and energy balance components
${\mathrm{\Delta T}}_{s,\mathrm{obs}}$	°C	Surface temperature change from radiometric tower estimated based on Stefan‐Boltzmann law
Ecophysiology components		
WUE	g C kg H_2_O^−1^	Water use efficiency
$G_{a}$	m s^−1^	Bulk aerodynamic conductance
$G_{s}$	m s^−1^	Surface conductance
*Ω*	Unitless	Surface—atmosphere decoupling coefficient
*u*, u*	m s^−1^	Wind speed, friction velocity
*Δ*	Unitless	Slope of saturated water vapor pressure curve against air temperature
γ	kPa K^−1^	Psychrometric constant
$C_{p}$	J kg^−1^ K^−1^	Specific heat of air at constant pressure
ρ	kg m^−3^	Air density
Other variables		
DTW	Unitless	Dynamic time warping
EF	Unitless	Evaporative fraction
Tair	°C	Air temperature
*LAI*	m^2^ m^−2^	Leaf area index
*NDVI*	Unitless	Normalized Difference Vegetation Index
*GCC*	Unitless	Green Chromatic Coordinates
${\mathrm{NIR}}_{v}$	Unitless	Near infrared reflectance of terrestrial vegetation index

The site is managed and continuously utilized for livestock grazing at a low density of 0.3 cows ha^−1^ (similar at all sites). During the driest months of summer (e.g., July to September) the cattle are usually relocated to nearby mountain grasslands. From 2004 to 2019, this site experienced a mean annual air temperature (Tair) of 16.7°C ± 0.64°C and received approximately 650 ± 177 mm of precipitation annually, with nearly 85% of the precipitation typically occurring between October and May. The dominant wind directions are west‐southwest and east‐northeast (Figure S7) (El‐Madany et al. 2018). This terrain here is mostly flat, with elevations ranging from 239 m to 285 m (Figure S6). The soil in this ecosystem is classified as Abruptic luvisol with a sandy upper layer (~5% clay, 20% silt, 75% sand between 0 and 20 cm) and a clay layer between 30 and 60 cm with low nutrient availability (Nair et al. 2019). Detailed information on soil nutrient content is available in Table S3.

**FIGURE 2:**
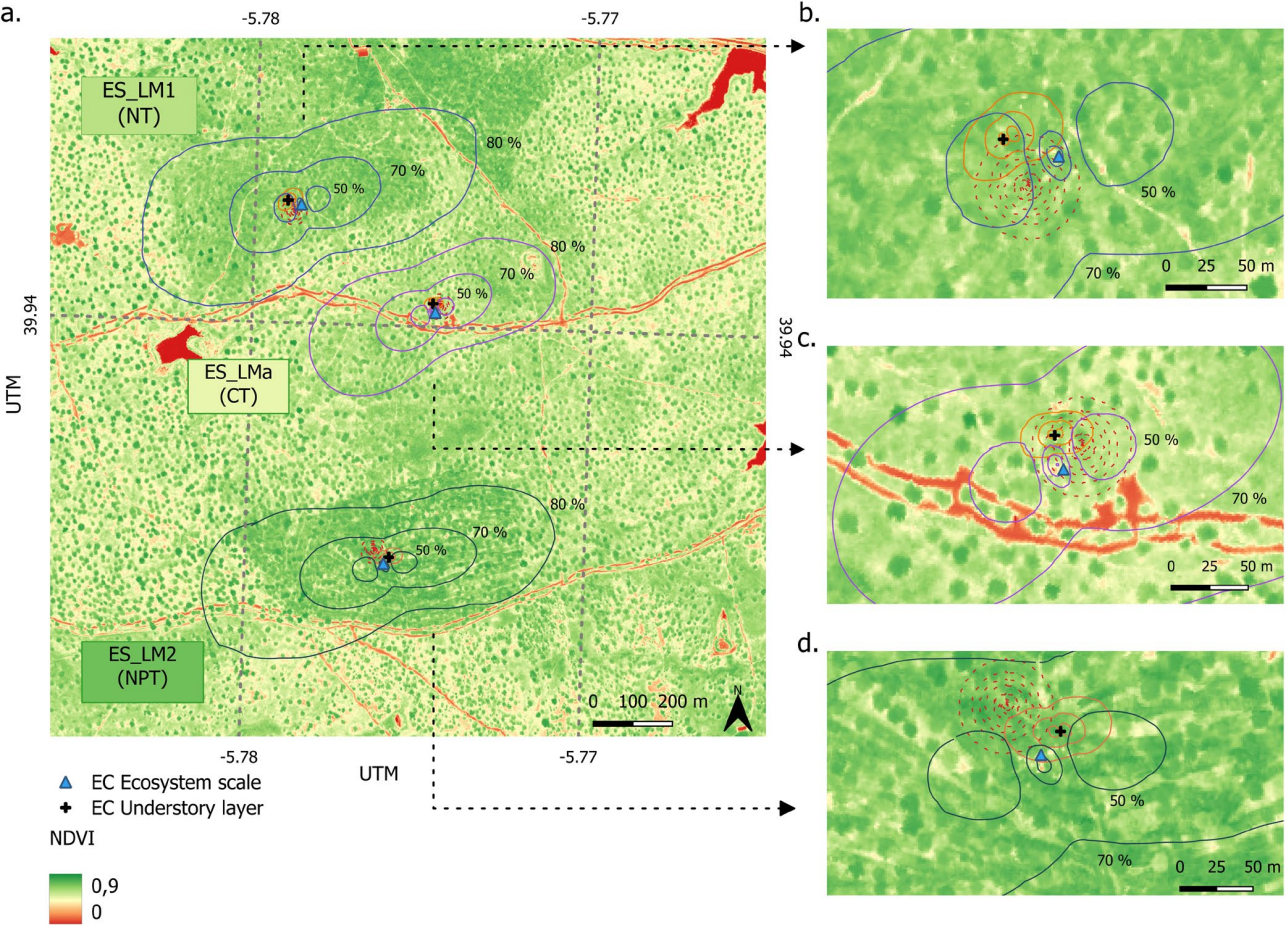
Footprint climatology for ecosystem scale eddy‐covariance (EC) towers (CT in dark purple is the control tower, NT in blue has nitrogen only addition, and NPT in black receives nitrogen and phosphorus addition) and in comparisons with understory EC footprints (orange) at Majadas de Tiétar. The background map is normalized difference vegetation index (NDVI) derived from the airborne flight campaign during spring 2015 (see Supplementary Method 1.1). This map is using Universal Transverse Mercator (*UTM*) as a map projection. The left column (a) illustrates all footprints with detailed isolines (50%, 70% and 80%) labelled for ecosystem scale EC footprints. The right column zooms in for the understory footprint climatology (orange line) and radiometric field of view (FOV) (dashed‐red line) inside the ecosystem scale EC footprint for NT (b), CT (c), and NPT (d). Blue triangle and black cross represent ecosystem and understory EC towers, respectively. Map lines delineate study areas and do not necessarily depict accepted national boundaries.

At Majadas de Tiétar, the control tower (CT) representing an area without nutrient addition, identified as ES‐LMa in FLUXNET (39° 56′ 25″ N, 5° 46′ 29″ W), and has been operated since 2003. In March 2014, two additional 15‐m height flux towers were established and a large‐scale nutrient experiment was initiated. North tower was designed to have N‐only addition (ES‐LM1 in FLUXNET, hereafter NT) (39° 56′ 33″ N, 5° 46′ 43″ W), positioned about 450 m northwest of CT. South tower has been receiving N+P addition (ES‐LM2 in FLUXNET, hereafter NPT) (39° 56′ 4″ N, 5° 46′ 33″ W), located 630 m in the southern direction from CT (Figure 2). Under the most frequent meteorological conditions, the footprints of the three ecosystem‐scale towers do not intersect (El‐Madany et al. 2018). The N and P nutrient additions were applied annually at similar times at each site, with decreasing amounts, except for some instances due to weather or logistic restrictions (e.g., pandemic) (Table S4, Nadolski et al. (2025)).

### 2.2 Eddy Covariance Data

Dual‐layer EC systems were established to continuously monitor land‐atmosphere interaction of both ecosystem and understory scales. For measuring turbulent fluxes, a sonic anemometer (SA‐Gill R3‐50; Gill Instruments Limited, Lymington, UK) was used to record three‐dimensional wind components and sonic temperature, along with an enclosed‐path infra‐red gas analyzer (LI‐7200; LI‐COR Bioscience, Lincoln, Nebraska, USA) to measure CO_2_ and water vapor (H_2_O) mixing ratios. These measurements were taken at a consistent height of 15 m above the ground (El‐Madany et al. 2021) to capture ecosystem‐scale signals. Additionally, understory EC towers were installed in November 2015, utilizing the same models of ultrasonic anemometer and gas analyzer, but positioned at a height of 1.6 m above the ground. These understory towers, located in open areas, are intended to monitor turbulent fluxes from the understory layer at all three sites, positioned approximately 30 m north of each ecosystem EC tower.

EC and meteorological data were collected and calculated as described in El‐Madany et al. (2018). The raw high‐frequency data were processed at 20 Hz using EddyPro v.7.0.9 (Fratini and Mauder 2014). To handle spikes, all raw data were despiked according to Vickers and Mahrt (1997) with linear interpolation, the arithmetic mean was removed from time series, and CO_2_ time lags were determined by covariance maximization in predefined windows. Spectral corrections included the analytical correction for high‐pass and low‐pass filtering effects (Moncrieff et al. 2004, 1997). For the two main wind directions (east‐northeast and west‐southwest), the planar fit method (Wilczak et al. 2001) was used for coordinate rotation, while double rotation was adopted for the other wind directions.

The CO_2_ storage flux was computed according to Aubinet et al. (2001) and corrected utilizing seven‐point profile measurements (0.1, 0.5, 1, 2, 5, 9, 15 m) (LI‐840; LI‐COR Bioscience) (Perez‐Priego et al. 2017). A friction velocity (u*) threshold was determined following Papale et al. (2006), and data with u* values below the established threshold were excluded from further analysis. Missing and low‐quality data (QC values > 1 according to Mauder and Foken (2011)) were filled using marginal distribution sampling following Reichstein et al. (2005). On average (2014–2023), the data coverage was 52.63 ±12.35%, 47.57% ± 5.90%, and 46.95% ± 6.54% for CT, NT, and NPT, respectively, after all the cleaning and filtering steps. Afterward, NEE were partitioned into gross primary productivity (GPP) and ecosystem respiration (Reco) according to Reichstein et al. (2005). Additionally, no energy imbalance correction was applied in the analysis. All post‐processing tasks were executed using the REddyProc package (Wutzler et al. 2018). Detailed information of gap‐filled NEE, quality flag, and partitioning results (i.e., daily GPP and Reco) can be found in Figures S13–S15.

### 2.3 Radiometric and Biometeorological Data

At each ecosystem‐scale flux tower, we also installed a CNR4 sensor (Kipp & Zonen, Delft, the Netherlands) to measure the incoming and outgoing shortwave (*SWDR* and *SWUR*) and longwave (*LWDR* and *LWUR*) radiations, enabling the calculation of net radiation (Rn). Beside each ecosystem EC tower, a standalone radiometric tower was built in November 2015 to monitor radiation components above a tree canopy and the understory layer (Figures 2 and S1). This radiometric tower uses the same model (CNR4), featuring a rotating arm that moves every 15 min (approximately 45° rotating angle) and records measurements at 5‐min measurement intervals (Figures S1 and S2). Additionally, these radiometric towers are also equipped with another CNR4 sensor fixed at a height of 3 m (without rotation) above the understory layer. To enhance understanding of energy budgets, each site is also equipped with eight soil heat flux (G) plates (HP3/CN3 Rimco, McVan Instruments, AUS) positioned 5 cm below the surface, with four plates located in open land and four under the tree canopy (established in November 2015). We calculated ecosystem G based on the weighted mean of the eight sensors (four in open land and four placed below canopy) following the 20% tree canopy cover.

EC and radiometric infrastructures provide measurements of CO_2_ fluxes, energy fluxes at both understory and ecosystem scales. Then, we used the radiometric data to estimate the ${\mathrm{Ts}}_{\mathrm{obs}}$ based on the Stefan‐boltzmann law:

(1) $$\text{LWUR}=\sigma \varepsilon {\mathrm{Ts}}_{\mathrm{obs}}^{4}$$

In this equation, 𝝈 denotes the Stefan‐boltzmann constant, and 𝜺 represents surface emissivity. The surface emissivity (𝜺) is approximated by an empirical relationship with albedo as 𝜺 = −0.16𝜶 + 0.99 (Juang et al. 2007).

Prior to the first nutrient manipulation, there was no significant difference in NEE observed between towers during the spring of 2014 (Figure S4). Afterward, at both NT and NPT, the annual NEE transitioned from being a weak CO_2_ source to a CO_2_ neutral (El‐Madany et al. 2021). The comparison of the near infrared reflectance of terrestrial vegetation index (${\mathrm{NIR}}_{v}$) from hyperspectral airborne images during pre‐ and post‐nutrient addition also showed a similar pattern to that of NEE. Detailed calculation of ${\mathrm{NIR}}_{v}$ and comparison vegetation properties during pre‐ and post‐nutrient addition can be found in Supplementary Method 1.1 and Figure S3. In addition, despite the footprints of EC systems and radiometric measurements represent different patches and vary in sizes (Figure 2), both measurement systems have a very similar composition of herbaceous layer and trees. It was observed that the FOV areas of the radiometers differ among the towers, and the potential biases from this mismatch in quantifying the surface α have been assessed (Figure S5).

### 2.4 Seasonal Variability Based on Phenological Transition Dates

In this study, the seasonality is determined based on phenological transition dates using in situ Green Chromatic Coordinates (GCC) captured from a digital camera (Stardot NetCam 5MP, StarDot Technologies). This digital camera was installed at the top of each ecosystem EC tower (15 m height), facing in the northern direction and captured images at 30‐min intervals. The cameras were established following the protocol of the PhenoCam network (https://phenocam.nau.edu/webcam/tools/). GCC represents vegetation greenness and computed as fraction of the green digital numbers with total of red, blue, and green (Richardson et al. 2009). We selected a region of interest within the understory layer at each site to determine phenological transition dates following Luo et al. (2018). The images and GCC data are available on the Phenocam network (site ID: ES_LM1 (NT), ES_LMa (CT), and ES_LM2 (NPT)) with a specific mask for the understory layer (GR_1000). Five phenological seasons (e.g., autumn, winter, spring, drydown, and summer) were defined following Nair et al. (2024) (Figure S16).

### 2.5 Data Analysis

#### 2.5.1 Radiative Forcing Analysis

We evaluated RF from the change of the surface α $\left({\mathrm{RF}}_{∆\alpha }\right)$ and cumulative NEE effect $\left({\mathrm{RF}}_{∆\mathrm{NEE}}\right)$ following Graf et al. (2023) and Ney et al. (2019). The net RF due to ∆NEE and ∆α were calculated using differences between the nutrient added sites and control tower (e.g., Δ = NT − CT or NPT − CT) on a monthly timescale. The process of filtering and quality check of NEE data is explained in section 2.2, while α underwent a quality check described in Supplementary Method 1.3. For surface α, we choose a specific time period only at midday (11:00–14:30) with a high clear sky index (> 0.7) and excluding rainy days to minimize the shadowing impact. Here we calculate surface as ratio of outgoing to incoming of shortwave radiation (i.e., SWUR/SWDR).

To estimate ${\mathrm{RF}}_{\text{ΔNEE}}$, the fluctuations in global atmospheric CO_2_ dry mole fraction following the local CO_2_ sinks or sources can be formulated as follows:

(2) $$∆\chi_{{\mathrm{CO}}_{2}}=\frac{\beta \times ∆\mathrm{NEE}\times t\times A_{\text{site}}\times M_{a}}{m_{a}\times M_{c}}$$

where *t* represents time, $A_{\text{site}}$ denotes the surface area of the ecosystem, $m_{a}$ is the mass of the atmosphere (5.15 × 10^21^ g), $M_{a}$ and $M_{c}$ are the molar masses of air and C, respectively. Those values required if NEE is expressed in g C per area and time ($\frac{M_{a}}{M_{c}}=2.414$), and β represents an estimate of the airborne fraction (0.44) (Graf et al. 2023). Then the ${\mathrm{RF}}_{\text{ΔNEE}}$ could be approached following Myhre et al. (1998):

(3) $${\mathrm{RF}}_{∆\mathrm{NEE}}=5.35\times \ln\left(1+\frac{∆\chi_{{\mathrm{CO}}_{2}}}{\chi_{0,{\mathrm{CO}}_{2}}}\right)$$

where 5.35 is an empirical value in W m^−2^ and $\chi_{0,{\mathrm{CO}}_{2}}$ is the base concentration of CO_2_ in the atmosphere (approximately 399.4 ppm in 2014). Considering $∆\chi_{{\mathrm{CO}}_{2}}\ll$ $\chi_{0,{\mathrm{CO}}_{2}},$ Equation (3) can be linearized as:

(4) $${\mathrm{RF}}_{∆\mathrm{NEE}}=5.35\times \left(\;\frac{∆\chi_{{\mathrm{CO}}_{2}}}{\chi_{0,{\mathrm{CO}}_{2}}}\right)$$

When bringing surface properties (α and SWDR) into a term of back radiation at TOA, an additional correction is required to adjust atmospheric absorption. Therefore, surface and atmosphere radiation were substituted by model‐derived data, known as surface α kernels (Bright and O'Halloran 2019). In this study, the calculation of ${\mathrm{RF}}_{\Delta \alpha }$ used publicly available radiative kernel models: CAM5 (Pendergrass et al. 2018), HadGEM2 (Smith et al. 2018), HadGEM3‐GA7.1 (Smith et al. 2020), and CACK 1.0 (Bright and O'Halloran 2019). These kernel models were compared, and their ensemble mean was computed to represent surface feedback at TOA (Graf et al. 2023). All kernels exhibited a strong correlation (*r* ^2^ ≥ 0.91) with measured SWDR from ecosystem scale (Figure S17). Then, the global ${\mathrm{RF}}_{\Delta \alpha }$ of a local surface α change, neglecting any longwave radiation feedback can be approached as (Bright and O'Halloran 2019):

(5) $${\mathrm{RF}}_{\Delta \alpha }=-K_{\alpha_{s}}\times ∆\alpha_{s}\times \frac{A_{\text{site}}}{A_{\text{Earth}}}$$

where $K_{\alpha_{s}}$ is ensemble mean of radiative kernels, $∆\alpha_{s}$ is the difference of albedo between the nutrient‐added and control sites, and $A_{\text{Earth}}$ is the surface area of the earth (5.1 × 10^14^ m^2^). Due to linearization of ${\mathrm{RF}}_{\text{ΔNEE}}$ we can include $A_{\text{site}}$ into both Equations (4) and (5) to give us the global effect of each square meter of treated land surface. Accordingly, we can combine radiative forcing ($\Sigma_{\mathrm{RF}}$) from both ΔNEE and Δα as follow:

(6) $$\begin{array}{c} \sum_{\mathrm{RF}}=\frac{{\mathrm{RF}}_{m}}{A_{\text{site}}}≃\frac{-K_{\alpha_{s}}\times ∆\alpha_{s}}{A_{\text{Earth}}}+\sum_{i=1}^{m}\frac{5.35\times \beta \times ∆{\mathrm{NEE}}_{i}\times M_{a}}{\chi_{0,{\mathrm{CO}}_{2}}\times m_{a}\times M_{c}} \end{array}$$

where *m* denotes the month of surface change under consideration after nutrient addition. Therefore, ${\mathrm{RF}}_{m}$ represent the global RF in a monthly timescale. The unit of RF in this study is expressed as 10^−14^ W m^−2^ (global) m^−2^ (treated surface) following the definitions used in Ney et al. (2019) and Gnanamoorthy et al. (2021), to represent global impacts of the targeted non‐abrupt vegetation changes. A positive value of RF indicates the net warming effect, while a negative value represents the net cooling effect. This approximation of global impact of RF is only rough estimation and it is possible to have opposite warming or cooling effects on the surface (Rotenberg and Yakir 2010). We further converted ${\mathrm{RF}}_{\Delta \alpha }$ to equivalent CO_2_ emission using global warming potential (GWP) to make it comparable with estimates from the literature of α‐induced radiative forcing (Yu et al. 2024). The calculation of ${\mathrm{GWP}}_{\Delta \alpha }$ can be found in Supplementary Method 1.6.

#### 2.5.2 *ΔTs* Decomposition Analysis

To assess energy fluxes and their mechanisms in influencing ∆Ts, we implemented a framework of ∆Ts decomposition ($∆T_{s,\mathrm{cal}}$) through a first‐order Taylor series approximation, as described by Luyssaert et al. (2014).

(7) $$∆T_{s,\mathrm{cal}}≃\frac{1}{4\sigma \varepsilon T_{s}^{3}}\left[-\text{SWDR}\times ∆\alpha +\left(1-\alpha \right)∆\text{SWDR}+∆\text{LWDR}-∆\mathrm{LE}-∆H-∆G-∆I-\sigma T_{s}^{4}∆\varepsilon \right]$$

where I represents a residual imbalance (W m^−2^). In theory, this residual flux (I) includes: the errors due to neglecting photosynthesis fluxes, heat storage, and systematic and random errors from measuring radiative and energy fluxes (Luyssaert et al. 2014). Thus, in this study, I is not only linked to energy balance closure but also other systematic errors from the EC system. Detailed processes of $∆T_{s,\mathrm{cal}}$ decomposition is explained in Supplementary Method 1.2. We compared $∆T_{s,\mathrm{cal}}$ with $∆T_{s,\mathrm{obs}}$, which indicates the ΔTs obtained directly from the LWUR (Equation 1).

The ΔTs decomposition analysis was performed on a daily time scale using quality‐checked half‐hourly energy fluxes and meteorological data as described in Supplementary Method 1.3. We only included data where $∆T_{s,\mathrm{obs}}$ and $∆T_{s,\mathrm{cal}}$ had identical signs and their difference was below a 2°C threshold. We excluded rainy days and data with more than 66% of nighttime or 66% daytime measurement missing within a 24‐h period. Those conditions could lead to potential errors considering the limitation of EC systems capturing turbulent and energy fluxes under high moisture conditions (Paulus et al. 2024).

In addition, we also obtained tree canopy RF and performed ∆Ts decomposition using data from the rotating radiometric tower when FOV is above a single tree (Figure S2). Then, we linked it (i.e., RF and ∆Ts) with the warming or cooling at the ecosystem scale with tree sap flux data (Supplementary Method 1.4, SAPFLUXNET) to give us insights of tree canopy cooling capacity (Supplementary Method 1.5).

#### 2.5.3 The Role of Aerodynamic‐Surface Conductance and Water Use Efficiency

In order to investigate the relation of ecophysiological characteristics with nutrient addition, we also calculated WUE, bulk aerodynamic conductance ($G_{a}$), and surface conductance ($G_{s}$) at daily timescale. WUE (g C kg H_2_O^−1^) represents the relationship between C uptake and water consumption (Law et al. 2002), defined as the ratio between productivity (GPP, g C m^−2^ d^−1^) and evapotranspiration (kg H_2_O m^−2^ d^−1^) on a daily basis.

$G_{a}$ (m s^−1^) is one of the important factors influencing H as it reflects the role of height and structure of the canopy. $G_{a}$ can be determined using wind speed (u) and $u^{*}$, as described by Monteith and Unsworth (1990):

(8) $$G_{a}≃{\left(\frac{u}{{u^{*}}^{2}}+6.2{u^{*}}^{-\frac{2}{3}}\right)}^{-1}$$

Furthermore, $G_{s}$ (m s^−1^) is calculated by inverting the Penman–Monteith equation (Monteith 1965), utilizing the measured LE values as follow:

(9) $$G_{s}≃\frac{\mathrm{\gamma LE}G_{a}}{∆\left(R_{n}-G\right)+\rho C_{p}\mathrm{VPD}G_{a}-\mathrm{LE}\left(∆+\gamma \right)}$$

where *Δ* represents the slope of saturated water vapor pressure curve against air temperature, γ is the psychrometric constant (kPa K^−1^), $C_{p}$ is the specific heat of air at constant pressure (J kg^−1^ K^−1^), VPD as vapor pressure deficit (kPa), and ρ is air density (kg m^−3^). This $G_{s}$ contains information about both stomatal and soil resistance to evaporation (Li and Wang 2019; Zhang et al. 2020). In addition, we also estimated the canopy‐atmosphere decoupling coefficient (*Ω*) to capture the surface‐atmosphere relation (Jarvis and McNaughton 1986).

(10) $$\Omega ≃\frac{\epsilon +1}{\epsilon +1+\frac{G_{a}}{G_{s}}}$$

where $\epsilon =\frac{s}{\gamma }$ is a dimensionless coefficient with s being the slope of the saturation vapor pressure curve (Pa K^−1^), and γ is the psychrometric constant (Pa K^−1^). Values close to 0 indicate well‐coupled conditions between surface and the atmosphere, while value close to 1 means the opposite. Well‐coupled condition means similar conditions at the canopy surface compared to the atmosphere above the canopy (Jarvis and McNaughton 1986). The calculations of $G_{a}$, $G_{s}$, and *Ω* were conducted using the R package “bigleaf” (Knauer et al. 2018).

#### 2.5.4 Distance Measure for Similarity of *ΔTs* Decomposition and Statistical Analysis

To assess which radiation and energy variables have high synchronization with $∆T_{s,\mathrm{cal}}$ (Equation 7), we conducted a distance measure analysis—dynamic time warping (DTW) (Giorgino 2009). The DTW method evaluates the temporal similarity between $∆T_{s,\mathrm{cal}}$ with radiative (i.e., δα,δSWDR,δLWDR,δε) and non‐radiative components (i.e., δLE,δH,δG,δI). DTW illustrates an optimal match between two given time series in a similar time dimension (Lee et al. 2020). We used this analysis considering the importance of surface‐atmosphere heat transfer capacity and temporal variation over daily or seasonal cycles when estimating surface temperature from energy budget (Lohmann 2020). The wind speed and direction keep changing at sites, leading to mismatch in source area of surface energy components (Foken 2008; Gao et al. 2010). Those processes also further increase the difficulty to assess the driver of surface temperature change due to high surface‐atmosphere heat transfer dynamics and component with large magnitude in the ∆Ts decomposition might only exert small effect locally (e.g., near‐surface area). Therefore, in DTW analysis, each radiation‐ and energy‐related component was converted from the flux unit (W m^−2^) into degree temperature (°C) (symbolized with δ). To ensure the comparability of DTW values across all components, we normalized the DTW value. A lower DTW value indicates that a particular component closely resembles with $∆T_{s,\mathrm{cal}}$ temporally.

We also performed significance tests on differences between sites using either ANOVA or Kruskal‐Wallis test. We conducted that using CO_2_ flux (NEE), albedo (α), energy fluxes (i.e., LE,H,G,I), WUE, and ecophysiology parameters at a daily time scale following the data filtering and quality checks explained in Supplementary Method 1.3. The selection of ANOVA or Kruskal–Wallis was based on data distribution and homogeneity, followed by a post hoc test with an alpha of 0.05 as thresholds.

## 3 Results

### 3.1 Domination of Albedo Changes Over CO_2_ Uptake on Net Radiative Forcing in Response to Nutrient Manipulation

#### 3.1.1 Co‐Directional Effects of Albedo and CO_2_ Uptake on RF due to Non‐Abrupt Vegetation Change

**FIGURE 3:**
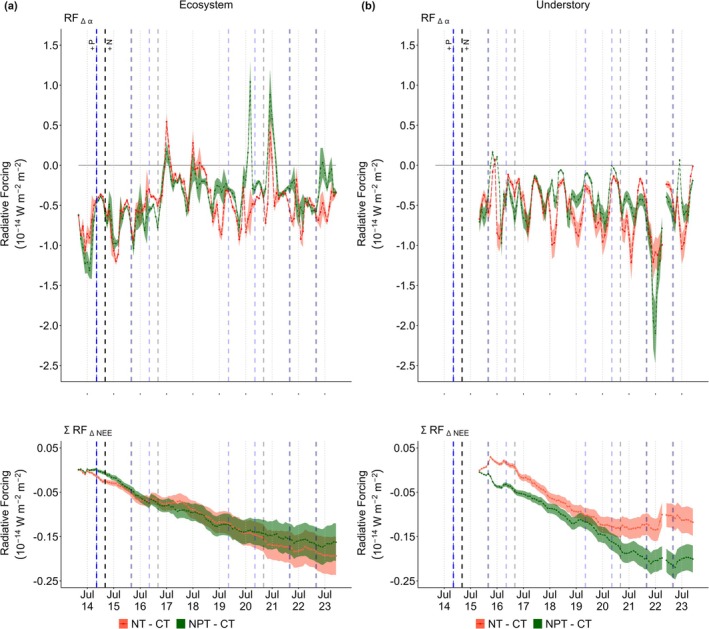
Monthly ecosystem (a) and understory (b) radiative forcing based on Δ*α* (${\mathrm{RF}}_{\Delta \alpha }$) and Δ NEE (${\mathrm{RF}}_{\text{ΔNEE}}$). ${\mathrm{RF}}_{\text{ΔNEE}}$ is a cumulative value while ${\mathrm{RF}}_{\Delta \alpha }$ is monthly value. The values represent global impact in 10^−14^ W m^−2^ (global) m^−2^ (surface). The vertical blue and black dashed lines represent the timing of P and N nutrients added, respectively. The red and dark green areas represent the standard deviation value of RF at NT–CT and NPT *–* CT, respectively.

While nutrient addition enhances CO_2_ uptake, its impact is considerably less pronounced than the influence of ∆α on RF (Figure 3). Notably, both ${\mathrm{RF}}_{\Delta \alpha }$ and ${\mathrm{RF}}_{\text{ΔNEE}}$ contribute to the cooling effect with distinct patterns observed between the ecosystem and understory scales. Based on the changes on ecosystem scale, ${\mathrm{RF}}_{\Delta \alpha }$ demonstrated a stronger cooling effect at NT (−0.46 ± 0.08 10^−14^ W m^−2^ m^−2^) compared to NPT (−0.39 ± 0.09 10^−14^ W m^−2^ m^−2^). Initially, ${\mathrm{RF}}_{\Delta \alpha }$ at ecosystem level show a positive trend at the beginning of nutrient addition, then the pattern is relatively consistent after 3 years with a substantial warming (*p* > 0.05) effect during summer. Understory ${\mathrm{RF}}_{\Delta \alpha }$ also showed a similar pattern with ecosystem scale, NT had a lower value with an average of −0.54 ± 0.30 10^−14^ W m^−2^ m^−2^ compared to NPT (−0.47 ± 0.35 10^−14^ W m^−2^ m^−2^). However, there is a sharp decline in understory ${\mathrm{RF}}_{\Delta \alpha }$ at NPT in 2022, which was most likely caused by legacy effects of heat waves since 2019. This pattern did not sustain after 2 years and the high biomass from increased photosynthesis made NPT very bright (i.e., many senescent leaves) in 2022 (Figure S25), leading to the sharp drop in ${\mathrm{RF}}_{\Delta \alpha }$.

In contrast to the pattern of ${\mathrm{RF}}_{\Delta \alpha }$, understory ${\mathrm{RF}}_{\text{ΔNEE}}$ revealed a stronger cooling effect at NPT (−0.12 ± 0.07 10^−14^ W m^−2^ m^−2^) compared to NT (−0.08 ± 0.05 10^−14^ W m^−2^ m^−2^). During 2020–2021, NPT managed to have less CO_2_ emissions at the understory due to increased photosynthesis. Thus, the understory ${\mathrm{RF}}_{\text{ΔNEE}}$ of NT and NPT separated from each other after 2020, but both plateaued after 2022. ${\mathrm{RF}}_{\text{ΔNEE}}$ of NT and NPT stay consistent at about −0.10 and −0.20 10^−14^ W m^−2^ m^−2^, respectively, afterwards. The pattern of understory ${\mathrm{RF}}_{\text{ΔNEE}}$ is also linked to the reverse WUE pattern between NT and NPT in response to nutrient addition (Figures 9b and S25c). These results further emphasize the domination of cooling effect from ∆α especially at NT, while NPT showed stronger ∆NEE in the understory.

#### 3.1.2 Nutrient Addition Enhances CO_2_ Uptake and Increases Surface Albedo

**FIGURE 4:**
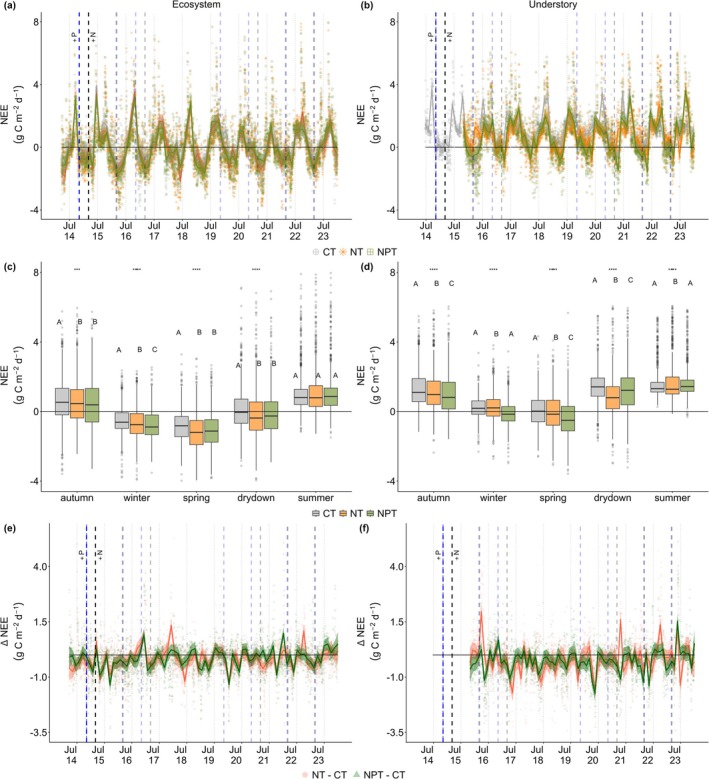
Time series of daily net ecosystem exchange (NEE) of ecosystem (the left column) and understory (the right column) scales across seasons and sites (CT—control, NT—nitrogen [N] only addition, and NPT—nitrogen and phosphorus [P] addition). The vertical blue and black dashed lines represent the timing of P and N nutrients added, respectively. The grey, orange, and light green lines and dots represent the daily NEE (a, b), while the boxplot summarizes the seasonal daily NEE (c, d) of *CT*, *NT*, and *NPT*, respectively. Meanwhile, the red and dark green lines and dots on e and f represent daily ∆NEE, while the shaded areas reflect uncertainty for NT− CT and NPT− CT, respectively. Asterisks on the top of each boxplot (c, d) represent significant difference with *p < 0.05, **p < 0.01, ***p < 0.001, and ****p < 0.0001. Letters on the boxplot represent post‐hoc HSD test, the same letter meaning not significantly different (post hoc HSD, *p* < 0.05). Dots on the boxplot represents outlier, horizontal line inside the box is the median value, while the box shows the interquartile range of the data distribution.

**FIGURE 5:**
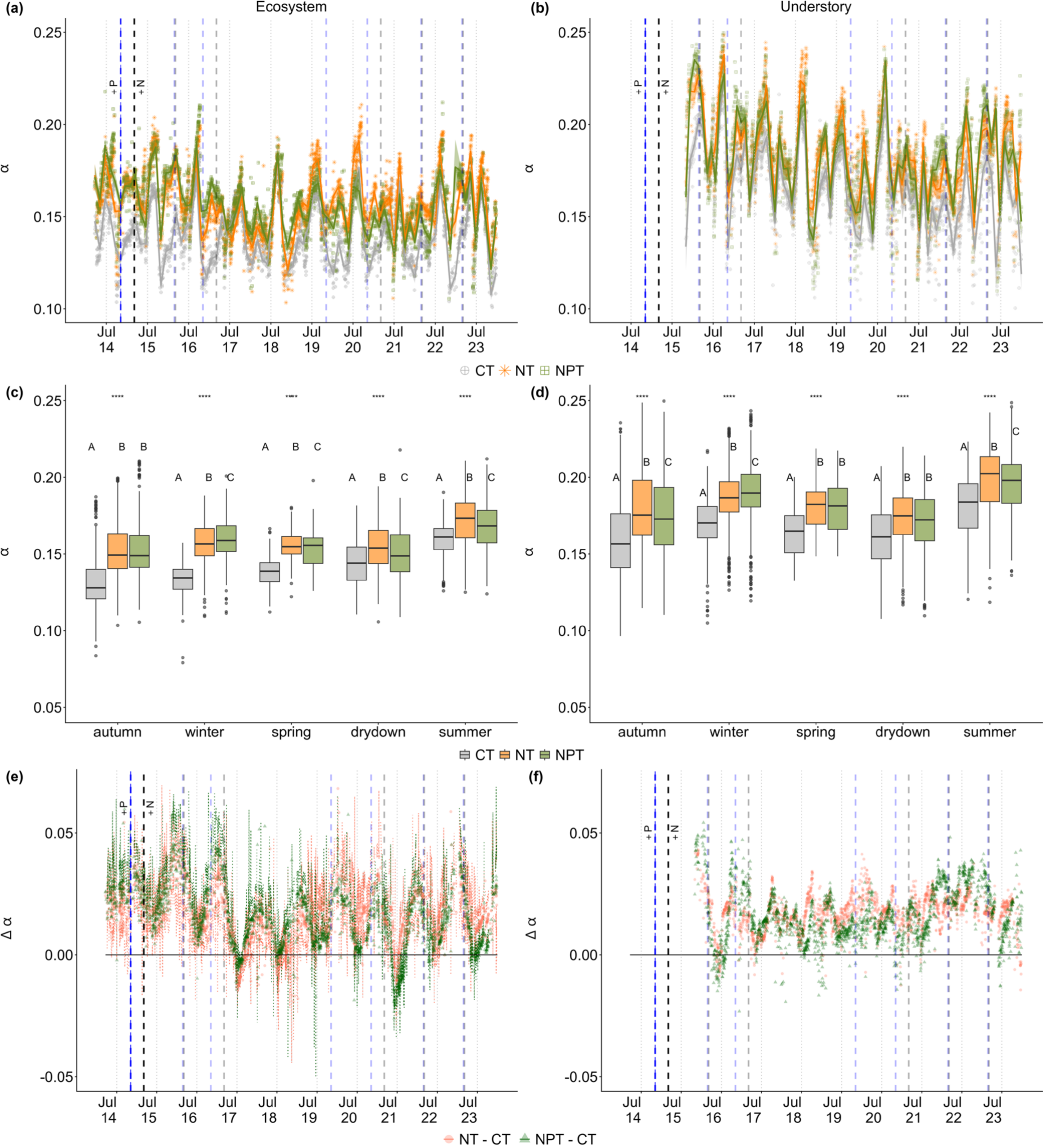
Time series of daily midday α at ecosystem scale (the left column) and understory (the right column) across seasons and sites (CT—control, NT—nitrogen only addition, and NPT—nitrogen and phosphorus [P] addition), including: daily midday α (a–d), and daily midday ∆α (e and f). The dotted lines in e represent the uncertainty based on midday α linear regression between sites before nutrient addition started (see Figure S5). The vertical blue and black dashed lines represent the timing of P and N nutrients added, respectively. The red and dark green lines represent the daily ∆α, while the shaded areas reflect uncertainty for NT− CT and NPT− CT, respectively. Asterisks on the top of each boxplot (c, d) represent significant difference with *p < 0.05, **p < 0.01, ***p < 0.001, and ****p < 0.0001. Letters on the boxplot represent post‐hoc HSD test, the same letter meaning not significantly different (post hoc HSD, *p* < 0.05). Dots on the boxplot represents outlier, horizontal line inside the box is the median value, while the box shows the interquartile range of the data distribution.

In semi‐arid regions, nutrient addition enhances CO_2_ uptake (Figure 4), but it also increases the surface α (Figure 5). However, the seasonality of NEE and surface α vary between the ecosystem and understory. For instance, during summer, no substantial difference in daily NEE was observed between sites at the ecosystem scale (*p* > 0.05), whereas on understory layer, a significant difference was noted at NT − CT in the same season (*p* < 0.05). During growing period (e.g., spring), daily understory ∆NEE indicates a stronger CO_2_ sink at NPT (mean ± SD: −0.53 ± 0.89 g C m^−2^ d^−1^) than NT (−0.13 ± 1.09 g C m^−2^ d^−1^) (Figure 3f, *p* < 0.05). The strong increases in understory productivity at NPT are also reflected by the highest GPP compared to CT and NT (Figure S14). Different than the understory layer, no substantial difference was observed between nutrient manipulation treatments (NT and NPT) at the ecosystem scale across most of the seasons (Figure 4c, *p* > 0.05). This contrasting response of nutrient addition between ecosystem and understory scales is linked to a stronger increase in N concentration on the understory over tree layer (Figure S10). These results also further emphasize the dominant role of understory layer on daily NEE.

Similar to ∆NEE patterns, understory ∆α also showed a noticeable response of nutrient addition compared to ecosystem scale. The strong response on understory layer is indicated by higher ∆α value (NT − CT: 0.017 ± 0.007 and NPT−CT: 0.015 ± 0.011) compared to ecosystem scale (NT − CT: 0.015 ± 0.010 and NPT−CT: 0.013 ± 0.012). In relation to CO_2_ uptake, there is a higher correlation between leaf N concentration and NEE at NPT (*r* = −0.56, *p* < 0.05) compared to NT (*r* = −0.14, *p* > 0.05), leading to a slightly less positive understory NEE, especially during growing periods (Figure S11a). However, there are two different correlations between understory leaf N concentration and α at the two nutrient addition sites, with NT: showed more neutral (*r* = 0.02, *p* > 0.05), while a negative correlation was found at NPT (*r* = −0.22, *p* > 0.05) (Figure S11c). These findings underscore the distinct responses of nutrient manipulation captured at the leaf and understory scales.

### 3.2 A Scale Dependent of Warming and Cooling Effects

#### 3.2.1 Dissimilarity Between Surface Temperature Change and Radiative Forcing

**FIGURE 6:**
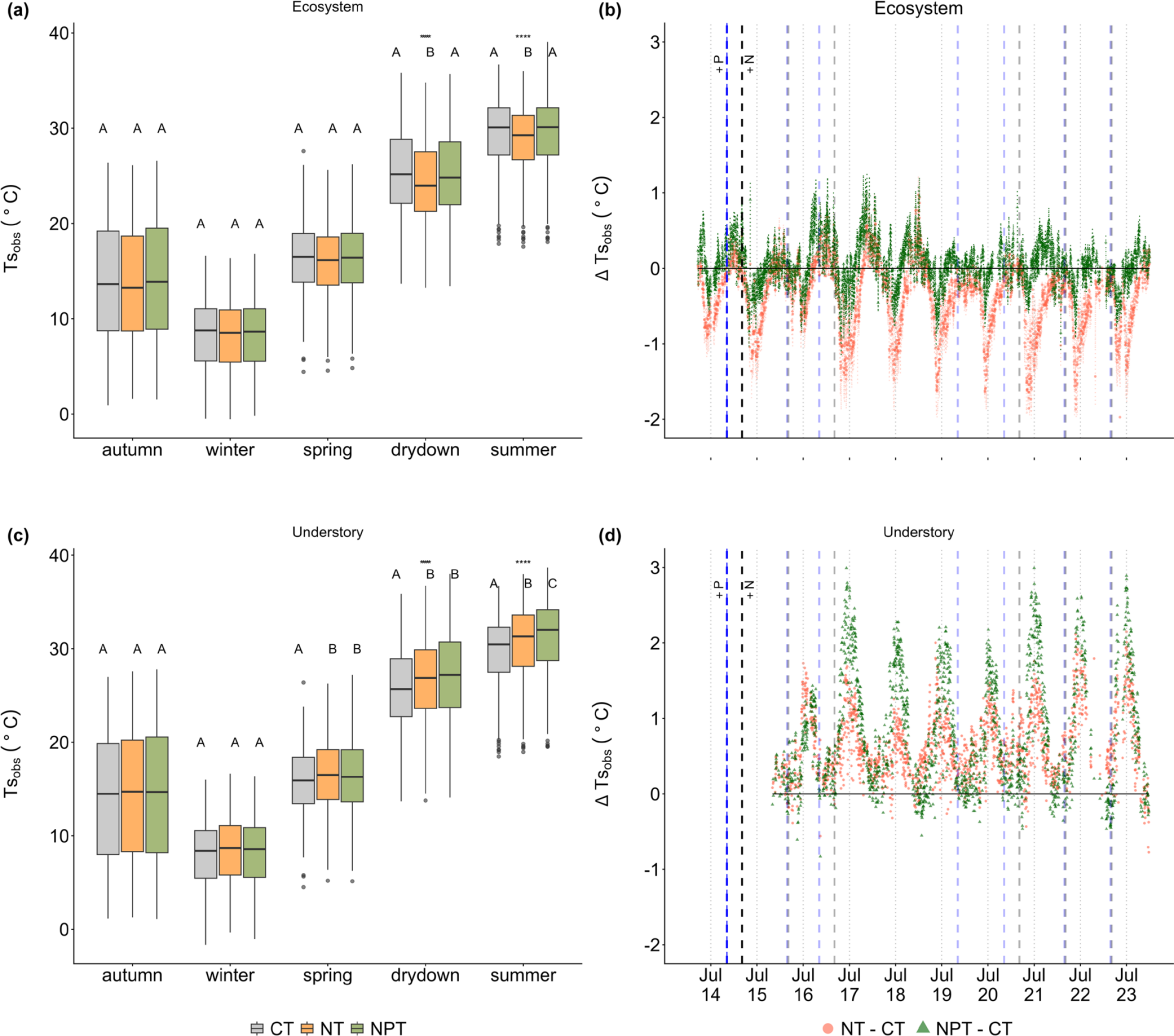
Daily surface temperature (${\mathrm{Ts}}_{\mathrm{obs}}$) at ecosystem (the left column) and understory (the right column) scales across seasons and sites (CT—control, NT—nitrogen [N] only addition, and NPT—nitrogen and phosphorus [P] addition), including mean daily Ts (a and c) derived using data from radiometric sensor (Equation 1) and differences of daily Ts ($∆{\mathrm{Ts}}_{\mathrm{obs}}$) between sites (b and d). The vertical blue and black dashed lines represent the timing of P and N nutrients added, respectively. The red and dark green lines represent the daily ${\mathrm{Ts}}_{\mathrm{obs}}$ for NT− CT and NPT− CT, respectively. Asterisks on the top of each boxplot (a, c) represent significant difference with *p < 0.05, **p < 0.01, ***p < 0.001, and ****p < 0.0001. Letters on the boxplot represent post‐hoc HSD test, the same letter meaning not significantly different (post hoc HSD, *p* < 0.05). Dots on the boxplot represents outlier, horizontal line inside the box is the median value, while the box shows the interquartile range of the data distribution.

Different patterns can be identified between $∆T_{s,\mathrm{obs}}$ and the cooling effect observed through ${\mathrm{RF}}_{\Delta \alpha }$ and ${\mathrm{RF}}_{\text{ΔNEE}}$. Notably, only NT at ecosystem scale exhibit a similar cooling effect ($∆T_{s,\mathrm{obs}}$: −0.41°C ± 0.47°C), while NPT at ecosystem scale relatively have a slight difference with average $∆T_{s,\mathrm{obs}}$ of 0.03°C ± 0.28°C compared to CT (Figure 6). Although there are significant differences found on daily understory NEE among sites during autumn and spring (*p* < 0.05) (Figure 4d), the same patterns are not observed in ${\mathrm{Ts}}_{\mathrm{obs}}$ (*p* > 0.05) (Figure 6c). In contrast, the significant differences in daily understory ${\mathrm{Ts}}_{\mathrm{obs}}$ among sites are found during summer (*p* < 0.05). During summer, understory layer demonstrates significantly warmer $∆T_{s,\mathrm{obs}}$ at the nutrient‐added sites, with a higher value at NPT (0.80°C ± 0.77°C) compared to NT (0.63°C ± 0.46°C) (*p* < 0.05). These observations display the complex mechanisms across interfaces represented using NEE, surface α, and ${\mathrm{Ts}}_{\mathrm{obs}}$.

#### 3.2.2 The Dynamics of Energy Flux Between Ecosystem and Understory

**FIGURE 7:**
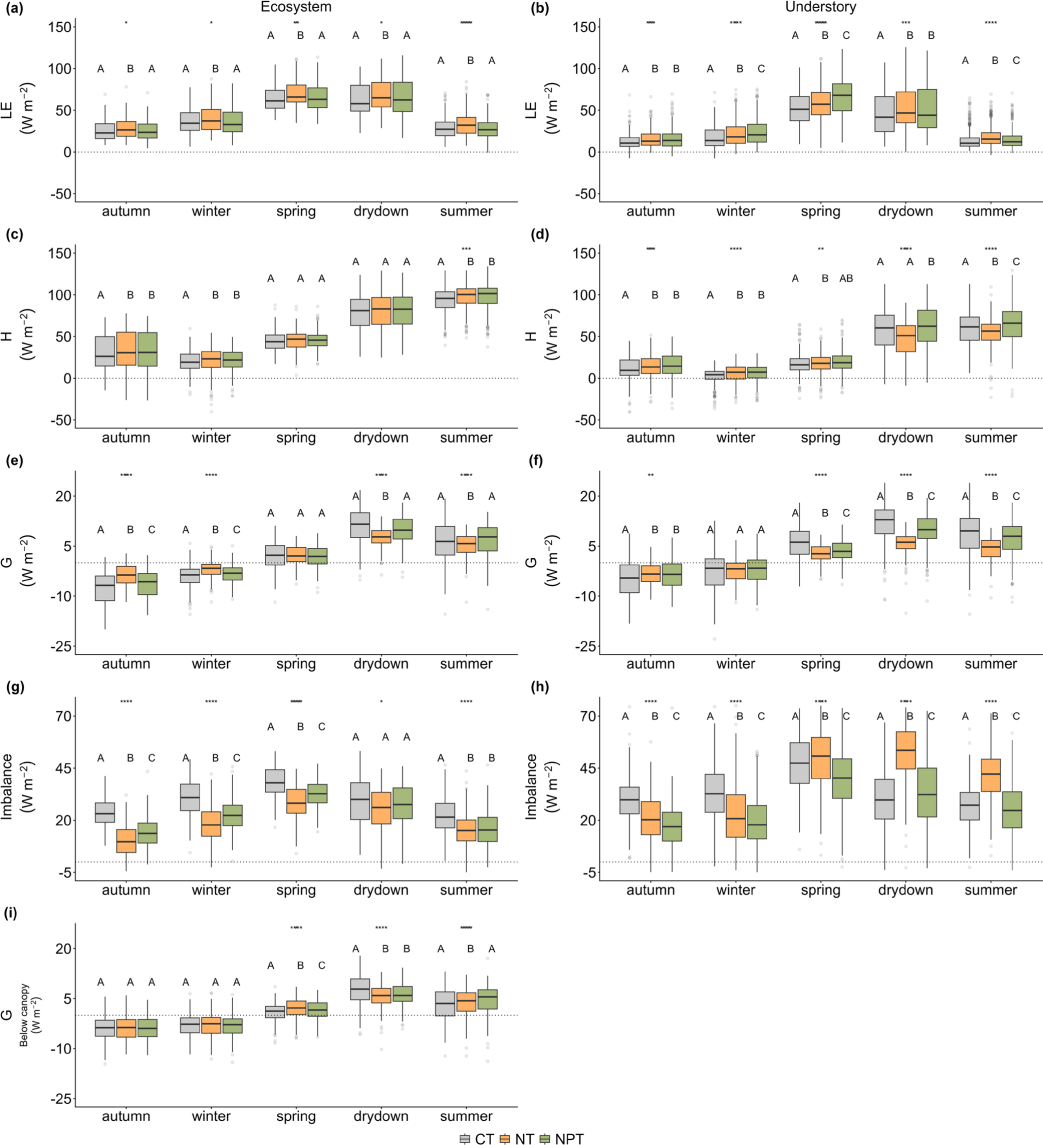
Mean daily of energy fluxes (i.e., LE, H, G and I) at ecosystem (a, c, e, g) and understory (b, d, f, h) scales, as well as daily G below canopy (i) across seasons and sites (CT—control, NT—nitrogen only addition, and NPT—nitrogen and phosphorus addition). Asterisks on the top of each boxplot (a, c) represent significant difference with *p < 0.05, **p < 0.01, ***p < 0.001, and ****p < 0.0001. Letters on the boxplot represent significant differences between sites, the same letter meaning not significantly different (post hoc HSD, *p* < 0.05). Dots on the boxplot represents outlier, horizontal line inside the box is the median value, while the box shows the interquartile range of the data distribution.

The dissimilar responses of $∆T_{s,\mathrm{obs}}$ to the nutrient treatments between ecosystem and understory scales reflect the complex mechanisms associated with the surface energy fluxes partitioning. This energy partitioning effect is more pronounced at the understory than ecosystem scale (Figure 7). At the ecosystem scale, NT shows the highest daily LE throughout the seasons (*p* < 0.05), whereas at the understory, NPT has the highest LE (*p* < 0.05), especially during growing periods. Furthermore, while nutrient addition led to an increase in daily H at the ecosystem scale, the rise was not significant (*p* > 0.05) (Figure 7c). However, at the understory, daily H at NPT shows the highest value (*p* < 0.05), particularly during water‐limited periods (i.e., drydown and summer). These are also observed in contrasting patterns of daily ET and evaporative fraction (EF), where NT shows the highest value compared to other sites at ecosystem scale (Figure S19a,b), while NPT experiences the highest value at the understory (Figure S19d).

The different patterns of daily G between ecosystem and understory scales further highlight potential insulation provided from denser herbaceous layers, attributed to a higher LAI, especially during growing seasons (i.e., spring) (Table S5). Additionally, the linkage between G and other surface energy balance partitioning (e.g., H) is only noticeable on the understory layer, especially during water‐limited periods (e.g., drydown and summer). During those periods, understory G and H showed a similar pattern, with NT having the lowest value. Those patterns partially translate to understory I but in the opposite direction, as NT showed the highest values compared to other sites. These dynamics highlight that, at the understory scale, less active vegetations during water‐limited periods make G and H more coupled but also lead to higher I, indicating more energy is not measured by the flux system.

#### 3.2.3 *ΔTs* Decomposition Capture the Dynamics of Radiation and Energy Flux Components in Response to Nutrient Imbalance

**FIGURE 8:**
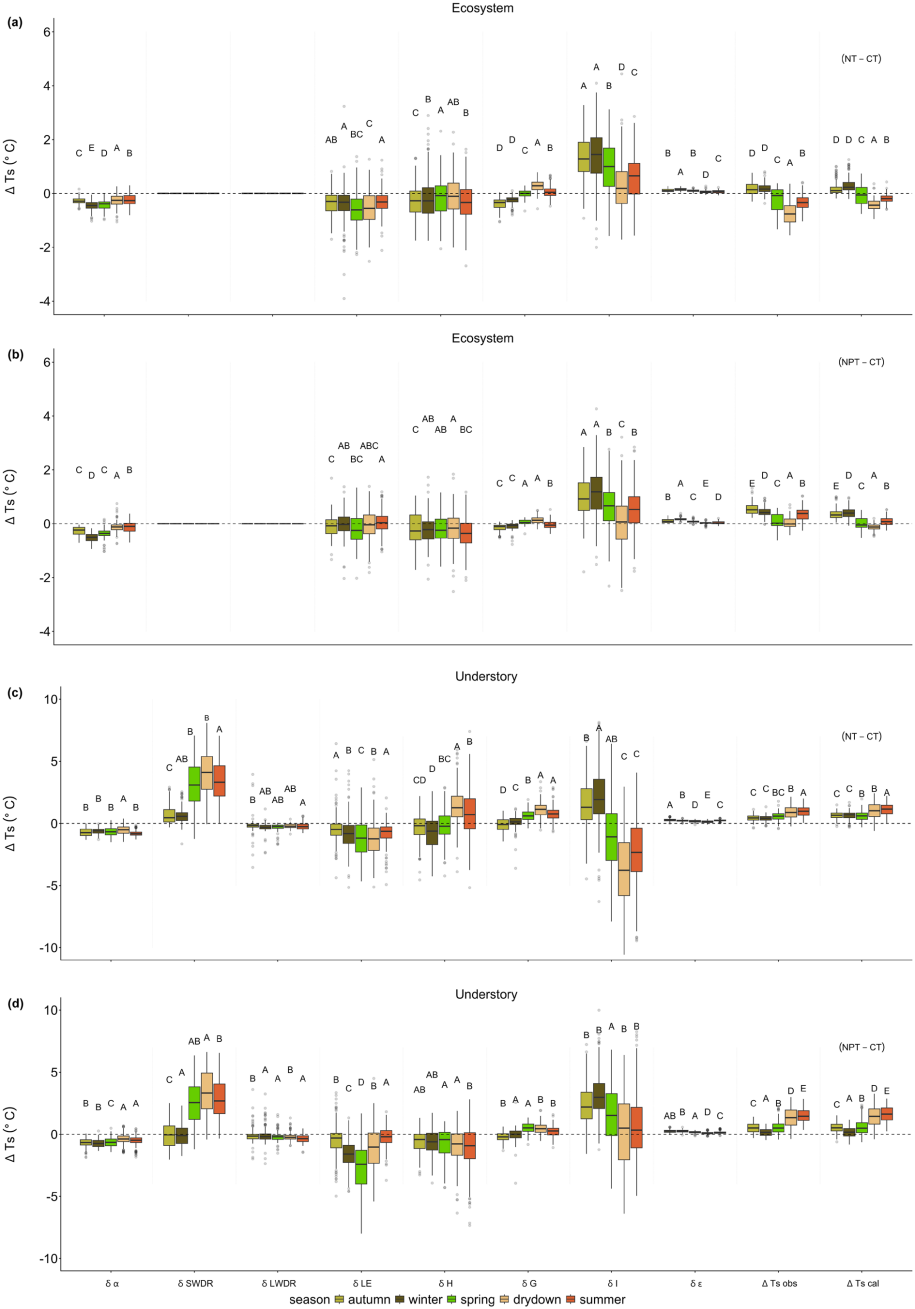
∆Ts decomposition based on changes in radiation and energy fluxes following nutrient manipulation at the ecosystem (a, b) and understory (c, d) scales across seasons and sites (CT—control, NT—nitrogen only addition, and NPT—nitrogen and phosphorus addition). δ denotes energy‐related components after converting the unit into degree temperature (from W m^−2^ to °C). Letters on the boxplot represent significant differences between season; the boxplot shared the same letter means not significantly different (post hoc HSD, *p* < 0.05). Dots on the boxplot represent outliers, horizontal line inside the box is the median value, while the box shows the interquartile range of the data distribution.

The degree of synchronization of energy fluxes partitioning (ΔLE,ΔH,ΔG, and ΔI) to the $∆T_{s,\mathrm{cal}}$ is more pronounced at the understory compared to ecosystem scale (Figure 8). At the ecosystem scale, δLE and δH at NT show slightly lower $∆T_{s,\mathrm{cal}}$ (on average at −0.40°C and −0.22°C, respectively) compared to NPT (on average at −0.04°C and −0.26°C, respectively). Meanwhile, at the understory, although δLE consistently leads to cooling effects (NT: −0.84°C and NPT: −1.03°C) along with δα (NT: −0.68°C and NPT: −0.57°C), these cooling effects are insufficient to counterbalance the warming influence from δSWDR (NT: 2.29°C and NPT: 1.71°C) and δG (NT: 0.47°C and NPT: 0.16°C). The differences in δSWDR at the understory scale are mainly due to the shadow effect from tree canopy development at the respective sites. It emphasizes the important role of tree canopy in semi‐arid ecosystems. Furthermore, the relationship of δH to the $∆T_{s,\mathrm{cal}}$ at understory also vary with nutrient addition. For example, NT exhibits a slightly stronger warmer effect due to δH (0.19°C ± 1.67°C on average), while NPT shows cooler $∆T_{s,\mathrm{cal}}$ (δH: −0.85°C on average) at the understory. This contrasting pattern further highlights the complex land‐atmosphere interaction mechanisms at the understory interface in response to the nutrient addition.

The complex near‐surface mechanisms of $∆T_{s,\mathrm{cal}}$ are further supported by the consistent high synchronization of understory δG to $∆T_{s,\mathrm{cal}}$ throughout all seasons, surpassing the shared variability from δSWDR. Meanwhile, at the ecosystem scale, subsequent with δα and δG, δLE ranked as the third most temporally‐aligned variables with $∆T_{s,\mathrm{cal}}$ (Table 2). Looking into details on seasonal pattern at understory, δLE on NPT demonstrates a close similarity with $∆T_{s,\mathrm{cal}}$ during summer, highlighting the higher heat transfer capacity during periods of reduced photosynthetic activity. After getting into the re‐greening season, the increased CO_2_ uptake (Figure 4) leads to the boosted cooling capacity from δLE, which shows the strong coupling between C and water. The results of DTW analysis also highlight that some seasonal $∆T_{s,\mathrm{cal}}$ values are greater due to the high synchronize temporal drivers also having larger magnitudes (i.e., δ LE and δ SWDR).

**TABLE 2:** Top three components with the highest temporal similarity with surface temperature differences (∆Ts in mean ± standard deviation) following the nutrient additions throughout the seasons. CT, NT, and NPT are the control, nitrogen only added, and nitrogen and phosphorus added sites.

		Autumn	Winter	Spring	Drydown	Summer		Autumn	Winter	Spring	Drydown	Summer
Ecosystem	*ΔTs* (°C)	−0.05 ± 0.28	−0.03 ± 0.23	−0.47 ± 0.44	−0.90 ± 0.42	−0.62 ± 0.29	*ΔTs* (°C)	0.22 ± 0.25	0.12 ± 0.25	−0.12 ± 0.26	−0.19 ± 0.25	0.03 ± 0.22
	*NT – CT*	δ *ε* (0.02)	δ *ε* (0.03)	δ *G* (0.03)	δ *α* (0.02)	δ *α* (0.03)	*NPT – CT*	δ *ε* (0.02)	δ *ε* (0.04)	δ *ε* (0.02)	δ *ε* (0.01)	δ *ε* (0.02)
		δ *α* (0.05)	δ *G* (0.08)	δ *ε* (0.03)	δ *G* (0.05)	δ *G* (0.05)		δ *LE* (0.06)	δ *G* (0.07)	δ *G* (0.02)	δ *α* (0.02)	δ *G* (0.03)
		δ *LE* (0.06)	δ *LE* (0.10)	δ *α* (0.03)	δ *LE* (0.06)	δ *LE* (0.07)		δ *G* (0.07)	δ *LE* (0.08)	δ *α* (0.03)	δ *G* (0.02)	δ *α* (0.05)
Understory	*ΔTs* (°C)	0.32 ± 0.28	0.38 ± 0.24	0.59 ± 0.41	0.88 ± 0.51	0.94 ± 0.40	*ΔTs* (°C)	0.34 ± 0.39	0.14 ± 0.26	0.56 ± 0.56	1.34 ± 0.78	1.49 ± 0.52
	*NT – CT*	δ *ε* (0.10)	δ *ε* (0.14)	δ *G* (0.09)	δ *G* (0.15)	δ *G* (0.19)	*NPT – CT*	δ *ε* (0.09)	δ *ε* (0.10)	δ *G* (0.08)	δ *G* (0.26)	δ *G* (0.29)
		δ *SWDR* (0.15)	δ *SWDR* (0.15)	δ *ε* (0.12)	δ *ε* (0.28)	δ *ε* (0.41)		δ *G* (0.14)	δ *G* (0.12)	δ *LWDR* (0.11)	δ *LWDR* (0.37)	δ *LE* (0.49)
		δ *G* (0.18)	δ *G* (0.20)	δ *LWDR* (0.19)	δ *LWDR* (0.35)	δ *LWDR* (0.43)		δ *LWDR* (0.17)	δ *α* (0.18)	δ *ε* (0.11)	δ *ε* (0.40)	δ *SWDR* (0.50)

*Note:* A lower value in brackets means the component is temporally closer to ∆Ts dynamics. See Table S2 for a complete DTW value for all ecophysiology components.

#### 3.2.4 The Dynamic of Surface‐Aerodynamic Conductance and Ecophysiological Properties Further Emphasize the Complex Mechanism Between Ecosystem and Understory

**FIGURE 9:**
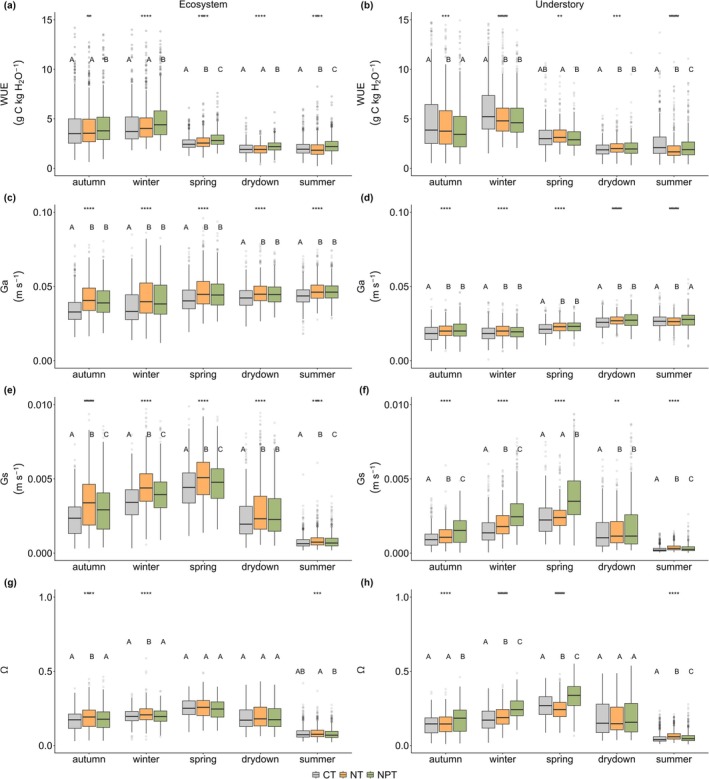
Physical and physiological properties at the ecosystem scale (the left column) and understory scale (the right column) across seasons and sites (CT—control, NT—nitrogen only addition, and NPT—nitrogen and phosphorus addition), including: mean daily water use efficiency (WUE) (a and b), mean daily aerodynamic ($G_{a}$) (c and d), mean daily surface conductance ($G_{s}$) (e and f), and mean daily omega decoupling (*Ω*) (g and h). Letters on the boxplot denote significant differences between sites (post hoc HSD, *p* < 0.05). Asterisks on the top of each boxplot (a, c) represent significant difference with *p < 0.05, **p < 0.01, ***p < 0.001, and ****p < 0.0001. Dots outside the boxplot represent outliers, horizontal lines inside the boxes are the median values, and the box borders show the interquartile ranges of the data.

Nutrient addition affects not only the surface α and energy partitioning, but also influences ecophysiological properties such as WUE, $G_{a}$, $G_{s}$, and *Ω*, which also exhibits different patterns between the ecosystem and understory scales (Figure 9). At the ecosystem scale, NPT has the highest WUE (3.38 ± 2.03 g C kg H_2_O^−1^) compared to NT (3.02 ± 1.85 g C kg H_2_O^−1^) and CT (3.05 ± 1.93 g C kg H_2_O^−1^), particularly during autumn and winter (Figure 9a). However, in the understory, an increasing WUE is only observed in spring (Figure 8b), while other seasons show a decrease in WUE. For example, CT demonstrates higher WUE (4.15 ± 3.72 g C kg H_2_O^−1^) than NT (3.61 ± 2.75 g C kg H_2_O^−1^) and NPT (3.52 ± 2.46 g C kg H_2_O^−1^) at the understory during autumn.

Furthermore, $G_{a}$ and $G_{s}$ collectively regulate heat exchange between surface and the atmosphere. Seasonal patterns of daily $G_{a}$ are similar but with different magnitudes between the ecosystem and understory scales (Figure 9c,d). At the ecosystem scale, nutrient addition leads to significant increases in daily average $G_{a}$ across the seasons (*p* < 0.05), with NT (0.045 ± 0.01 m s^−1^) being similar to NPT (0.044 ± 0.01 m s^−1^). At the understory, no significant difference is found in daily $G_{a}$ between NT (0.023 ± 0.005 m s^−1^) and NPT (0.024 ± 0.006 m s^−1^) (*p* > 0.05), although both were significantly different to CT (0.022 ± 0.006 m s^−1^) in most seasons except for summer (*p* < 0.05).

Unlike the consistent pattern of $G_{a}$ across ecosystem and understory scales, $G_{s}$ exhibits different patterns for the two layers (Figure 9e,f). At the ecosystem scale, nutrient addition leads to an increase in $G_{s}$, with NT constantly having the highest value (0.0031 ± 0.002 m s^−1^) when compared to NPT (0.0027 ± 0.002 m s^−1^) and CT (0.0024 ± 0.001 m s^−1^). On the other hand, at the understory, $G_{s}$ at NPT demonstrates the highest values (0.0019 ± 0.001 m s^−1^) compared to NT (0.0014 ± 0.001 m s^−1^) and CT (0.0011 ± 0.001 m s^−1^). These results strongly indicate $G_{s}$ is sensitive to nutrient addition and emphasize the contrasting heat transfer and capacity between the understory (near the ground surface) and ecosystem scales (influenced by the tree canopy). Furthermore, both ecosystem and understory EC exhibit low decoupling (Figure 9g,h), indicating well coupled condition between surface and the atmosphere. The low decoupling further emphasizes that the differences in RF and Ts are primarily due to complex mechanisms between energy flux partitioning and radiation components.

## 4 Discussion

### 4.1 The Role of Biophysical and Biogeochemical Changes due to Altered Nutrient Levels in Net Radiative Forcing

#### 4.1.1 The Domination of Albedo‐Driven Over CO_2_ ‐Uptake on Net RF as Impact of Non‐Abrupt Vegetation Change at Semi‐Arid Savanna

Our research highlights the domination of α‐driven biophysical feedback over biogeochemical effect represented by CO_2_ flux in determining the net RF. This domination is attributed to the fact that changes in surface α could instantaneously alter the energy balance, leading to immediate climatic effects (Beets 2000; Bright, Myhre, et al. 2015), whereas C cycle feedback becomes effective over longer timescales (Ahlswede et al. 2022; Graf et al. 2023). In this study, the stronger influence of ${\mathrm{RF}}_{\Delta \alpha }$ over ${\mathrm{RF}}_{\text{ΔNEE}}$ also emphasizes the unique ecological and regional climate constraints of semi‐arid ecosystems. These ecosystems are characterized by an energy‐limited state during growth periods with sufficient water supplies (i.e., winter–spring) and become water‐limited when radiation is abundant (i.e., drydown–summer), with this strong seasonality significantly affecting the ecosystem's ability to continue CO_2_ uptake. This domination of α‐driven feedback is also in line with the result from a global mapping of net climatic impact, which revealed that 60% of the area across Mediterranean forests, woodlands, and scrub biomes would lead to a cooling effect, with 76% derived from substantial α effects (Hasler et al. 2024).

While N fertilization boosts productivity, it accelerates the decrease of soil water content in the top soil (Sardans and Penuelas 2013). This water scarcity can decrease green LAI (El‐Madany et al. 2018), alter root depth (Nair et al. 2019), and reduce metabolic activity (Sardans and Penuelas 2013). Especially during drydown period, soil water content in the top soil drops significantly due to increased ET (Battista et al. 2018; Luo et al. 2018). N fertilization can also alter species diversity (Soons et al. 2017), and likely selects for early‐senescing species (Wang and Tang 2019). This is supported by changes in the composition of the main plant community (e.g., grasses, forbs and legumes) between nutrient addition and control sites (Martini et al. 2019; Migliavacca et al. 2017). N fertilization increases the abundance of forbs but decreases the graminoids, which led to changes in leaf angle distribution resulting in earlier senescence and yellowing (Martini et al. 2019). In agriculture systems, N fertilization could also alter leaf traits (e.g., pigmentation, cell structure), promoting growth while increasing surface α (Ollinger et al. 2008; Yu et al. 2024). As grass layer senescens more quickly when the dry period comes, it resulting in a brighter surface (Luo et al. 2020). Those processes likely explain why the NT and NPT sites have higher α compared to CT (Figure 5).

Another key point from this study is the cooling effect of both ${\mathrm{RF}}_{\Delta \alpha }$ and ${\mathrm{RF}}_{\text{ΔNEE}}$ align in the same direction, which contrasts with the inverse relationship identified by Graf et al. (2023). Typically, ${\mathrm{RF}}_{\Delta \alpha }$ and ${\mathrm{RF}}_{\text{ΔNEE}}$ move in opposite directions when assessing the impact of land use and land cover change that involves direct vegetation conversion (Ahlswede et al. 2022; Gnanamoorthy et al. 2021; Kirschbaum et al. 2011; Ney et al. 2019). The similar cooling effects between α and NEE is observed only in agriculture land system where cover crops are applied (Genesio et al. 2020; Guardia et al. 2019; Lugato et al. 2020) or in the cultivation of C4‐type vegetation for bioenergy (Eichelmann et al. 2016). This aligned cooling effect is closely related to the impact of N‐addition, which leads to higher leaf reflectivity, as shown at a study of N and water manipulation in a semi‐arid steppe in China where increased CO_2_ uptake and α continued to rise with denser vegetation cover (Zhang et al. 2015).

#### 4.1.2 Comparisons RF as Impact of Non‐Abrupt Vegetation Change at Semi‐Arid Savanna With Other Types of Vegetation Change

Although ${\mathrm{RF}}_{\Delta \alpha }$ utilizes the radiative kernel from global circulation model (Bright and O'Halloran 2019), it is important to mention that RF in this study is a rough estimation of global effect. The quantification of effective net RF (e.g., complete combination of ${\mathrm{RF}}_{\Delta \alpha }$ and ${\mathrm{RF}}_{\text{ΔNEE}}$) would require a global circulation model run with exact spatiotemporal pattern and magnitude of all surface property changes (Graf et al. 2023). Nevertheless, ${\mathrm{RF}}_{\Delta \alpha }$ provides valuable insights into potential global TOA changes considering local snapshot dynamics related to land management. ${\mathrm{RF}}_{\Delta \alpha }$ is widely used to assess the climate effects of deforestation (ranging from −2.10 to −21.51 W m^−2^) or afforestation projects (ranging from 5.48 to 1.34 W m^−2^) (Jiao et al. 2017; Ney et al. 2019; Zhu et al. 2024). However, there is a limited study on the impact of non‐disrupt change (e.g., N or P decomposition) within natural ecosystems. In this study, the direction of aligned cooling and α‐driven domination following the nutrient addition is the main focus. Furthermore, to compare against with results from other investigations, we have to use other metric, and GWP provides an equivalent metric of climate impacts of greenhouse gasses emission (Zhu et al. 2024). The conversion of α‐driven biophysical impact of N addition at our Mediterranean savanna (i.e., NT) using GWP is much larger (−582.03 to −426.27 kg CO_2_e ha^−1^ yr^−1^, see Table S1) compared to N fertilizer in European croplands (−263.64 ± 455.32 kg CO_2_e ha^−1^ yr^−1^) during growing periods (Yu et al. 2024), yet it is less than the impact on European winter wheat (798.35–929.34 kg CO_2_e ha^−1^ yr^−1^) (Ceschia et al. 2010). The α‐induced CO_2_e flux in this study is comparable to the conversion from grassland into N‐fertilized switchgrass for bioenergy (−520 ± 40 kg CO_2_e ha^−1^ yr^−1^) (Abraha et al. 2021). But, to note, biophysical impacts differ among fertilizer types and depend on the period under consideration (e.g., growing, harvest periods, tillage management), making direct comparisons still challenging. In summary, research of impacts of N accumulation have primarily concentrated on agricultural systems (Lugato et al. 2020; Yu et al. 2024), highlighting the necessity to explore these impacts in other environments, such as semi‐arid savannas, which are highly sensitive to N deposition. The finding of a similar cooling effect from ${\mathrm{RF}}_{\Delta \alpha }$ and ${\mathrm{RF}}_{\text{ΔNEE}}$ is noteworthy when compared to other regions facing water‐nutrient limitations.

### 4.2 A Multi‐Scale Dependent of Warming and Cooling Effect in Response to Nutrient Addition

#### 4.2.1 Complex Mechanisms of Surface Temperature Changes at Dual‐Layer Ecosystem

Our study also highlights the different net cooling effect among the understory, ecosystem, and global scales. When surface changes are translated to the TOA, a cooling effect is observed, whereas warmer ∆Ts are found near the surface‐atmosphere interfaces (Figures 3 and 6). This phenomenon is attributed to the fact that closer to the surface, the influence of the radiative mechanisms is generally less pronounced compared to energy flux components (e.g., LE and G) (Table 2) (Abera et al. 2019; Bonan 2008). The understory microclimate is governed by a complex interplay between radiation and energy fluxes (Montejo‐Kovacevich et al. 2020), and in this study, it is further influenced by the differential impacts from changes in nutrient levels.

In semi‐arid ecosystems, adding nutrient not only directly increases leaf N content, but also alters vegetation structure, which results in a greater LAI value (Table S5). These changes enrich the chlorophyll content (González‐Cascón et al. 2016), and lead to a higher fraction of absorbed photosynthetic active radiation (Perez‐Priego et al. 2015). El‐Madany et al. (2021) strengthen those relationships by emphasizing a strong linear relationship between LAI and maximum CO_2_ uptake by vegetation. The strong relationship between LAI and photosynthetic activity is also linked to stomatal conductance and transpiration rates, which affect the cooling capacity through LE (Martini et al. 2022; Zhou et al. 2021).

The amount of nutrients added and the dynamic of water availability also play an important role in influencing biomass productivity (Luo et al. 2020). Given different WUE and soil water extraction rates, those combinations might prevent added nutrients from becoming biologically available (Lee et al. 2010). At our site, those chain mechanisms lead to different responses in interannual variability in biophysics (e.g., α, LE, and G) and biogeochemical cycle (e.g., CO_2_ fluxes), with the understory often controlling biogeochemical processes across the entire ecosystem (Nadolski et al. 2025). At the N‐only added site, N addition led to P limitation, likely to increase P uptake directly from the water source into the plant or enabling increased photosynthesis and boosting soil microbes to extract P from organic matter (El‐Madany et al. 2021). Differences in soil water content extraction in the topsoil led to substantial interannual variability in WUE, as they are associated with nutrient availability and stoichiometric imbalance. A reduced growing season length, resulting from faster senescence (Luo et al. 2020), can decrease carbon uptake, leading to different seasonal patterns of WUE.

In addition to influencing leaf N concentration and LAI, nutrient addition also affects the energy flux partitioning, leading to a significant difference (*p* < 0.05) in EF between the ecosystem and understory scale. At the ecosystem scale, NT demonstrates the highest EF, whereas NPT shows the highest EF at the understory (Figure S19). The variation in EF pattern is closely linked to changes of $G_{a}$ and $G_{s}$, which control surface‐atmosphere exchanges (Massmann et al. 2019) and subsequently affect Ts (Bright, Zhao, et al. 2015). N‐only addition leads to a higher $G_{a}$ at the ecosystem scale, reducing resistance and facilitating better heat transfer from surface to the atmosphere (El‐Madany et al. 2021). Meanwhile, a higher $G_{s}$ at the understory of NPT represents denser vegetation. Generally, an increase in $G_{s}$ promotes a cooling effect due to heightened evapotranspiration activity (Young et al. 2021). However, a denser herbaceous layer might also reduce heat transfer to the soil, as reflected in lower G, especially during dry period (Figure 7f), leading to more heat retained near the surface, trapped by the dense herbaceous layer. When drydown period begins, the understory herbaceous layer might reduce evaporative cooling but still partially insulates the soil, so a larger fraction of available energy can manifest as higher surface temperature rather than deep soil warming (Battista et al. 2018; Luo et al. 2020, 2018). This finding emphasizes the competing mechanisms between direct thermodynamic energy of radiation and the cooling capacity contribution from $G_{s}$, leading to ∆Ts anomalies (Hales et al. 2004). Furthermore, the understory layer typically experiences lower wind speeds, limiting the heat dissipation from the surface to the atmosphere (Butterworth et al. 2024).

N addition in this system not only mobilizes P via microbial processes under emerging P limitation, but also further accelerates soil drying and shortens the growing period. Those chain processes not only led to enhanced growing‐season transpiration but also advanced dormancy and surface warming (El‐Madany et al. 2021; Luo et al. 2020). Prior to the dry periods, increased LAI can lead to a denser herbaceous layer that might trap heat inside and slow moisture exchange, leading to nutrient‐driven shifts in WUE. Those produce a complex microclimate where the radiative signal looks cooler but the ground surface and near‐surface can still warm when the upper soil has dried.

Another important note, the soils in this particular ecosystem have low nutrient availability (Schnabel et al. 2013), and adding nutrients leads to higher soil respiration (Figure S12). This response is opposite to the meta‐analysis done by Janssens et al. (2010), which reported negative effect of N on soil respiration in temperate forest ecosystems. The high soil respiration at NT is also reflected in understory Reco at NT, which also shows the highest value during the growth period (e.g., spring) (Figure S15). We believe this is linked to the ecosystem's limited ability to store C in biomass, which could not balance the dynamic energy availability in soils in response to nutrient addition (Figure 7).

#### 4.2.2 The Role of Tree Canopy Cooling Capacity

Our results also indicate lower ∆Ts at the ecosystem scale compared to understory (Figure 6), emphasizing the significant role of tree canopy in cooling. Radiometric measurements taken above individual tree crown at each site reveal that NT exhibits a significant higher albedo and lower Ts compared to that of NPT (Figures S21–S23). Additionally, sapflux data also confirm that the tree transpiration is the highest at NT, whereas NPT shows the lowest tree transpiration among the sites (Figure S24; Poyatos et al. (2020)). Although nutrients primarily accumulate in the understory, trees can also absorb N, which concentrates in the leaves, thereby enhancing the photosynthetic capacity (Fleischer et al. 2013). The high N concentration in tree leaves at the study site was verified by tree leaf foliage analysis done by González‐Cascón et al. (2016). N‐only addition causes rapid water usage to acquire P to balance the increased N level, leading to higher transpiration (Luo et al. 2020).

### 4.3 Limitations and Future Implications

#### 4.3.1 Challenges and Limitations at Unique Semi‐Arid Savanna

The semi‐arid ecosystem appears to have a homogeneous surface at the ecosystem or larger scale, yet it exhibits significant heterogeneity on the understory layer (Burchard‐Levine et al. 2021; El‐Madany et al. 2018). Thus, the variations in the distribution of tree and grass contribute to increased uncertainty due to canopy shadow and surface patches (Li and Wang 2019). It also emphasizes that the vertical profile of Ts is influenced by temperature inversion and the competing processes of supply and demand under advection (Wang et al. 2024). Furthermore, the soil characteristics in this area have high spatial heterogeneity, affecting the representativeness of factors such as G and soil moisture in the EC flux footprint (Luo et al. 2018; Paulus et al. 2022). Although G covers both areas below‐canopy and open spaces, it still has non‐negligible uncertainty when evaluating energy fluxes.

Furthermore, the energy balance closure from understory EC systems is considerably lower, averaging between 58% and 70%, compared to the ecosystem‐scale EC tower (i.e., between 87% and 89%) (Figure S20). This low closure can systematically raise the biases of surface climate diagnostics by underestimating turbulent fluxes, heat distribution, nighttime heat retention, and convective transport errors (Butterworth et al. 2024; Masseroni et al. 2014; Mauder et al. 2024). In addition, issues related to neglected energy terms such as spectral loss, storage, and advection fluxes also influence the surface‐atmosphere interaction, which often cannot be separated (Wang et al. 2024) and emphasizes the scale‐dependent heat transfer in tree‐grass coexistence. Moreover, limitation of the EC technique in capturing turbulent and energy fluxes under high moisture conditions also increases the uncertainty, considering dew and fog formation in tree‐grass coexistence retains at near‐surface (Paulus et al. 2024).

Although N‐only addition enhances tree transpiration and increases the canopy's cooling capacity compared to N+P nutrient addition, evaluating the role of trees in this ecosystem remains challenging. As illustrated in Figures 7 and 8, tree cooling effect significantly influences ∆Ts at the ecosystem scale. While radiometric data from a single tree canopy provides insight into a tree's role in heat transfers, there is still a lack of understanding regarding how the entire tree canopy (~20 E% tree fraction) contributes to the tree‐grass coexistence ecosystem. Furthermore, with a dual‐layer structure of scattered oak trees and herbaceous understory, the differences in energy flux partitioning and the varying cooling effects between understory and ecosystem scale illustrate the need for more detailed vertical heat transfer models (Metzger 2018; Xu et al. 2018). Tree‐grass coexistence potentially creates a unique vertical coupling relationship, such as the understory dominance in daytime LE and H (Dubbert et al. 2014) and lower wind speeds near the surface, which limits heat dissipation (Andreu et al. 2018).

It is also important to note that since $G_{s}$ is quantified based on the Penman‐Monteith equation, a classic example of a “big‐leaf” or single layer canopy paradigm model, the ∆Ts anomaly is also related to the limitation of this model, which cannot capture the complexity in multi‐layer ecosystems (e.g., tree‐grass coexistence) (Bonan et al. 2021). A two‐source energy‐combination model would be more adequate as it differentiates energy balance or resistances between vegetation and soil (Kustas and Norman 2000; Shuttleworth and Wallace 1985); however, it remains challenging (Andreu et al. 2018). Recent studies have further developed the model with an integrated three‐sources energy balance model, which separates the energy terms for overstory, understory and soil layer (Burchard‐Levine et al. 2021). Additionally, it is also crucial to consider the influenced of surface patches in the partitioning of ET between soil and vegetation, which affects ∆Ts (Hirsch et al. 2018).

#### 4.3.2 Future Implications

This region is projected to experience drier conditions in the future, which substantially affects vegetation functions, influencing not only biophysical responses (e.g., senescence, α‐change) but also biogeochemical processes (e.g., CO_2_ fluxes). The N:P imbalance tends to deplete water more rapidly during the spring peak and drydown periods, thereby accelerating the grass layer's progression to dormancy and death (Luo et al. 2020). Given the potential for change in species diversity due to N:P imbalance, this might alter vegetation phenology and responses to climate change at both understory and ecosystem scales.

To accurately predict the resilience of semi‐arid savannas, future climate models need to incorporate complex multi‐layered processes involved in tree‐grass coexistence. The responses of these ecosystems to climate change‐induced stresses, such as increased aridity and N deposition, remain insufficiently explored. The different biophysics and biogeochemical responses at multi‐scale can lead to variations and uncertainties in climate model predictions, which can limit our understanding of future ecosystem resilience (Hirsch et al. 2018). These different responses at multi‐scale also highlight the importance to take into consideration of scale‐emergent processes. Resolving a larger fraction of small‐scale processes could improve the interpretability of climate prediction models (Schneider et al. 2024). Consequently, multi‐scale biophysical models are critical to improve the uncertainties related to the impact of CO_2_, N deposition, and elevated temperature due to climate change (Rosenzweig et al. 2014).

## 5 Conclusion

When evaluating the impacts of nutrient changes across multiple scales using this unique experiment, we found that both N and N+P additions lead to less positive or more negative NEE (i.e., cooling from a biogeochemical perspective); however, it is marginal when comparing to the dominating cooling effects of altered biophysical property (i.e., α) on the net RF. In semi‐arid ecosystems, where two layers of vegetation widely co‐exist, a significant difference in heat transfer should be carefully assessed. Our findings support this by revealing complex warming or cooling patterns at multiple scales which are also often scale‐dependent. At global scale (i.e., RF at TOA), adding nutrients in this ecosystem results in the net cooling effect (i.e., both ${\mathrm{RF}}_{\Delta \alpha }$ and ${\mathrm{RF}}_{\text{ΔNEE}}$ being negative) using surface changes at both ecosystem and understory scales. However, both treatments result in increased Ts at the local surfaces, particularly at the understory, reshaping ecophysiological interactions in this water‐ and nutrient‐limited environment. In addition, our results imply that N‐only increases (i.e., more widely happening due to N deposition) is more effective at enhancing tree cooling on ecosystem‐scale Ts compared to combined N+P addition, with a cost of higher water use. To sum up, this study underscores the importance of nutrient stoichiometry in regulating biophysical and biogeochemical processes in semi‐arid savannas, which shall be considered in ecosystem management and climate modeling.

## Author Contributions

**Bayu Hanggara:** conceptualization, investigation, formal analysis, writing – original draft; **Tarek El‐Madany:** validation, data curation; **Arnaud Carrara:** validation, data curation; **Gerardo Moreno:** validation, data curation; **Rosario Gonzalez‐Cascon:** validation, data curation, methodology; **Vicente Burchard‐Levine:** validation, data curation, methodology; **M. Pilar Martin:** validation, data curation; **Stefan Metzger:** validation, methodology; **Anke Hildebrandt:** supervision, validation, writing – review and editing; **Markus Reichstein:** funding acquisition, supervision; **Sung‐Ching Lee:** conceptualization, supervision, validation, writing – review and editing.

## Conflicts of Interest

The authors declare no conflicts of interest.

## Supporting information


**Data S1:** gcb70782‐sup‐0001‐Supinfo.pdf.

## Data Availability

The data that support the findings of this study are openly available in Zenodo at https://doi.org/10.5281/zenodo.15799672. In addition, ecosystem‐level data are available on the European Fluxes Database: https://www.europe‐fluxdata.eu/. PhenoCam data are available at https://phenocam.nau.edu/. To find the data on both the European Fluxes Database Cluster and the Phenocam network, site IDs are sufficient. The site IDs are: ES‐LMa (for the control tower, CT), ES‐LM1 (for the north tower, NT), and ES‐LM2 (for the south tower, NPT). Furthermore, radiative kernels data are publicly available at: CAM5 (https://zenodo.org/records/997902); HadGEM2 (https://doi.org/10.5518/406); HadGEM3‐GA7.1 (https://zenodo.org/records/3594673); and CACK 1.0 (https://doi.org/10.6073/pasta/d77b84b11be99ed4d5376d77fe0043d8).
