# Supplementary material for: Non‐Abrupt Vegetation Changes due to Altered Nutrient Balance Make Complex Scale‐Dependent Warming and Cooling Effects
Source: Glob Chang Biol. 2026 Mar 17;32(3):e70782. doi: 10.1111/gcb.70782 (PMC12993706; doi:10.1111/gcb.70782)
Supplement: Supplementary file 1 — Data S1: gcb70782‐sup‐0001‐Supinfo.pdf. [file GCB-32-e70782-s001.pdf]

Journal: Global Change Biology

Supplementary information

Non-abrupt vegetation changes due to altered nutrient balance makes complex scale-dependent warming and cooling effects

Hanggara B[1,2], El-Madany TS[1], Carrara A[3], Moreno G[4], Gonzalez-Cascon R[5], Burchard-Levine V[6], Martin MP[6], Metzger S[7,8], Hildebrandt A[2,9], Reichstein, M[1,2], and Lee, SC[1]

Contents

1. Methods 3
   1.1 Vegetation indices based on hyperspectral airborne images 3
   1.2 ΔTs decomposition equations 3
   1.3 Data filtering and quality checks for ΔTs decomposition 5
   1.4 Tree radiative forcing and ΔTs decomposition 6
   1.5 Tree transpiration using sapflux data 6
   1.6 Albedo-induced global warming potential ($GWP\Delta\alpha$) 7
   1.7 LAI and nutrient concentration sampling 7
   1.8 Soil respiration 8
2. Annual albedo-driven radiative forcing 9
3. DTW temporal similarity analysis 10
4. Measurement setup and pre-nutrient addition status 11
   4.1 Soil nutrient characteristic before nutrient addition 11
   4.2 Majadas measurement set up 11
   4.3 Radiometric tower set up 12
   4.4 Near infrared index for trees before and after nutrient addition 12
   4.5 Ecosystem NEE correlation between sites before nutrient addition 13
   4.6 Ecosystem albedo correlation between sites before nutrient addition for midday albedo uncertainty 13
5. Site terrain and windrose 14
   5.1 Digital elevation model map 14

5.2 Windrose for each EC tower 15
6. Timeline of nutrient addition 16
7. Leaf nutrient content and LAI 17
7.1 Herbaceous LAI 17
7.2 Herbaceous leaf nutrient concentration 17
7.3 Tree leaf nutrient concentration 18
7.4 Leaf N, C and C:N ratio 18
7.5 Leaf N and P correlation with NEE and albedo 19
8. Time series of soil respiration 19
9. Percentage of gap-filled NEE, quality flag and daily partitioning 20
9.1 Percentage of gap-filled 20
9.2 Time series daily GPP 21
9.3 Time series Reco 22
10. Seasonal characteristics of Majadas based on phenocam 23
11. Radiative kernel comparison with surface SWDR 24
12. Comparison between ΔTs observed from CRN4 sensor and ΔTs calculated 25
13. Evaporative fraction and evapotranspiration 25
14. Energy balance closure 26
15. Tree layer response on nutrient addition 27
15.1 Daily midday α and ΔTs on tree 27
15.2 Comparison ΔTs observation and ΔTs calculation on tree 28
15.3 Radiative forcing on tree 29
15.4 Tree sapflux transpiration 29
16. Summer albedo anomaly 30
16.1 Understory α during summer 30
17. References 30

1. Methods

1.1 Vegetation indices based on hyperspectral airborne images

Five high spatial resolution airborne hyperspectral images were processed over the Majadas experimental site. Hyperspectral flight campaigns were conducted before (08 April 2014) and after (23 April 2015, 03 July 2015, 03 May 2016, 19 May 2017) nutrient manipulation treatments. This flight campaign was operated by the Spanish National Institute for Aerospace Technology (INTA) and equipped with two sensors: Compact Airborne Spectrographic Imager (CASI-1500i; Itres Research Ltd., Canada) and Airborne Hyperspectral Scanner (AHS). The CASI (CASI-1500i) has a spatial resolution of 1.5 m and 144 spectral bands ranging from 0.38 to 1.05 µm (Burchard-Levine et al., 2021). At the same time, the AHS sensor has 80 spectral bands ranging from 0.43 to 13 µm with a spatial resolution of 4.5 m. For details about the processing of the hyperspectral images, we refer to Pacheco-Labrador et al. (2017).

We extracted ecosystem and grass vegetation index to test the pre- and post- nutrient addition treatment in this study. Normalized Difference Vegetation Index (NDVI) was calculated from CASI data following Rouse Jr et al. (1974). Here ρ is the hemispherical-directional reflectance factor for a specific wavelength in nm. The numbers denote the wavelengths.

$$NDVI = \frac{\rho 843 - \rho 666}{\rho 843 + \rho 666} \quad (1)$$

Then we calculate additional near infrared reflectance of terrestrial vegetation (NIRv) following Badgley et al. (2017).

$$NIR_v = \rho_{NIR} \times NDVI \quad (2)$$

1.2 ΔTs decomposition equations

To assess energy fluxes and their mechanisms in influencing ΔTs, we implemented a framework of ΔTs decomposition through a first-order Taylor series approximation (Luyssaert et al., 2014). Rn is calculated as the sum of the incoming and outgoing short- and long-wave radiation:

$$Rn = SWDR - SWUR + LWDR - LWUR \quad (3)$$

The relationship between $SWUR$ and $SWDR$ can be expressed using the equation $SWUR = \alpha \cdot SWDR$, where α is a factor. Considering all three sites were located in the same landscape, we

combined incoming short- and longwave radiation at the ecosystem scale. The same value of $SWDR$ and $LWDR$ across the landscape was also used to estimate ecosystem α. However, we keep $SWDR$ and $LWDR$ from CNR4 of grass layer for calculating ΔTs decompose at grass layer because of the consistent impact of tree canopy shadow. Subsequently, various turbulent energy fluxes utilizing Rn in their processes:

$$Rn = LE + H + G + I \tag{4}$$

Where LE denotes latent heat (Wm^{-2}), H is sensible heat (Wm^{-2}), G is soil heat fluxes (Wm^{-2}), and I represents a residual imbalance occurring in EC measurement due to energy balance non-closure (Foken, 2008). The residual imbalance (*I*) thus includes: the error due to neglecting photosynthetic fluxes, the error due to neglecting heat storage in the biomass and atmosphere, and systematic errors and random errors from measuring radiative and energy fluxes (Luyssaert et al., 2014). By combining equations (3) and (4), we can eliminate R_n to connect radiation and energy fluxes:

$$(1-\alpha)SWDR + LWDR - LWUR = LE + H + G + I \tag{5}$$

$LWUR$ is measured by CNR4 sensor but also could expressed as a function of Ts using the Stefan-Boltzmann law:

$$LWUR = \sigma\varepsilon T_{s,obs}^4 \tag{6}$$

In this equation, σ denotes the Stefan-Boltzmann constant, and ε represents surface emissivity. Surface emissivity (ε) is approximated by an empirical relationship with albedo as $\varepsilon = -0.16\alpha + 0.99$ (Juang et al., 2007). Thus, integrating equations (5) and (6) gives:

$$(1-\alpha)SWDR + LWDR - \sigma\varepsilon T_s^4 = LE + H + G + I \tag{7}$$

Using equation (9), we can estimate the full derivative of ΔTs decomposition through a first-order Taylor series approximation, as described by Luyssaert et al. (2014):

$$\Delta T_{s,cal} = \frac{1}{4\sigma\varepsilon T_s^3}[-SWDR \cdot \Delta\alpha + (1-\alpha)\Delta SWDR + \Delta LWDR - \Delta LE - \Delta H - \Delta G - \Delta I - \sigma T_s^4 \Delta\varepsilon] \tag{8}$$

$\Delta T_{s,cal}$ in equation (8) represents the calculated Ts reflecting contributions from radiative and energy fluxes components (Luyssaert et al., 2014; Zhang et al., 2020). Meanwhile, $\Delta T_{s,obs}$ indicates the surface temperature change obtained directly from the $LWUR$ using equation (6). This analysis was conducted on a daily time scale with quality-checked half-hourly energy fluxes and meteorological data as described in Supplementary Method 1.3. We only included data where $\Delta T_{s,obs}$ and $\Delta T_{s,cal}$ had identical sign and their difference was below a 2°C threshold. In addition, we also performed tree canopy RF and ΔTs decomposition on a single tree canopy using the radiometric tower and incorporated tree sap flux data (Supplementary method 1.4, SAPFLUXNET) to give us insight into tree canopy cooling capacity on each site.

In principle, there should be no $\Delta SWDR$ (i.e., SWDR difference between the towers) given that the sites are situated within a similar landscape. Nevertheless, our result revealed a significant $\Delta SWDR$ between nutrient addition sites and CT at the grass layer. This $\Delta SWDR$ is particularly evident in the grass layer due to the variation in tree crown area of each site, which resulted in differing canopy shadows and microtopography (See Supplementary Fig S1). Microtopography and canopy shadow can influence the diffuse radiation components and solar exposure (Weiser et al., 2016). The $\Delta SWDR$ also contributed to a stronger effect of ΔLE and ΔH at the grass layer, indicating that this layer is more dependent on radiation inputs and associated surface heat transfers (Juang et al., 2007).

1.3 Data filtering and quality checks for ΔTs decomposition

The analysis was conducted at the daily time scale with quality-checked half-hourly energy fluxes and related meteorological data as follows (Luyssaert et al., 2014):

a) Half-hourly measurements were averaged to apply the ΔTs decomposition at a daily resolution. A filter was applied to contain only days in which both day and night observations were present to get a representative daily mean. Twenty-four-hour periods with more than 66% of nighttime or 66% of daytime measurements missing were excluded.
b) The residual imbalance flux (I) was calculated daily. A regression analysis between (Rn-G) and (H+LE) was conducted to estimate values of I, with additional detrending to remove the seasonal pattern. All days with detrended values exceeding the 95% confidence interval were removed from the analysis.
c) An Additional filter was applied to remove outliers. The first and third quartiles were defined as high and low threshold values. Values 1.5 times above or below the threshold were discarded from the time series.
d) Surface albedo (α) is calculated as the ratio of SWUR to SWDR, measured at the ecosystem scale (15m height) and grass layer (3m height from the radiometric tower) at each site. Daily α was averaged from midday (11:00 – 14:30) to avoid high solar zenith angle uncertainty. We also filtered out cloudy days with clear sky index > 0.7, representing the ratio of SWDR to extraterrestrial radiation at the TOA (Wood et al., 2015). To minimize the impact of canopy shadow and microtopography at ecosystem scale, we enlarge the uncertainty of albedo value with linear regression on summer 2014 (pre-fertilization). During the summer of 2014, before the commencement of nutrient addition, a significant difference in midday α at the ecosystem scale (15m height) was observed between towers, assuming that the grass vegetation was fully senescent. We assumed this is due to the field of view from the CNR4 sensor at CT having a bigger impact from the tree canopy compared to NT and NPT (Supplementary Fig. S1). Therefore, this factor is accounted for by incorporating additional

uncertainty based on the linear regression slope when comparing α between nutrient-added sites (NT/NPT) and CT (Supplementary Fig. S4).

e) We exclude rain events (precipitation > 0 mm).

f) Finally, we only accept records when $\Delta T_{s,obs}$ and $\Delta T_{s,cal}$ have the same sign and the difference is less than a threshold of 2°C. A comparison between $\Delta T_{s,obs}$ and $\Delta T_{s,cal}$ can be found in Supplementary Fig S7.

In addition, we calculated the daily evaporative fraction (EF) of the ecosystem and the grass layer. EF is defined as the ratio between LE and available energy (EF = LE / (LE + H)) (Tong et al., 2022). EF can be utilized as a diagnostic variable for vegetation water status (Nutini et al., 2014), which has a strong relationship with soil moisture, VPD (Tong et al., 2022), and vegetation cover (Gentine et al., 2007). Daily and seasonal EF on the ecosystem and grass layer can be found in Supplementary Fig S8.

1.4 Tree radiative forcing and ΔTs decomposition

Tree radiative forcing and ΔTs decomposition followed similar data filtering and quality checks described in section Supplementary method 1.2. Radiation and albedo value of the tree layer derived from rotating the radiometric tower from a single tree canopy of each site (see Supplementary Fig. S1). Due to the absence of tree CO_2 flux monitoring, we define tree NEE (Net ecosystem exchange) following equation S1 based on flux footprint analysis done by El-Madany et al. (2018). The footprint area of ecosystem EC measurement in the three towers was similar, with the grass layer dominating (75 – 80% footprint area) compared to tree canopy (15 – 20%) and bare soil (< 5%).

$$NEE_{eco} = \sum NEE_{tree} \cdot f_{tree} + \sum NEE_{grass} \cdot f_{grass} = NEE_{tree} \cdot 0.2 + NEE_{grass} \cdot 0.8 \quad (9)$$

We used this flux fraction to determine the ΔNEE for calculating $RF_{\Delta NEE}$ from tree layer, while $RF_{\Delta\alpha}$ used α value from rotating radiometric tower (Supplementary Fig S1).

For the estimated ΔTs decomposition from tree layer, we combined the energy fluxes (LE, H, I) from radiometric tower into one variable due to the limitation on missing information on energy fluxes specific to a single tree canopy. We utilized soil heat flux (G) under tree canopy to calculate the energy flux variable based on the net radiation equation $R_n = LE + H + G + I$ (see Supplementary Fig. S12).

1.5 Tree transpiration using sapflux data

Tree transpiration used for compare the ΔTs from radiometric sensor with tree cooling capacity in ecosystem. Tree transpiration was estimated by the heat ratio method (SFM1 Sap Flow Meter, ICT

International) in six trees within the footprint of the ecosystem EC tower. The trees were selected based on the stem diameter at breast height (DBH). For details about the processing of the tree sap flux transpiration, we refer to (Perez-Priego et al., 2017). The sap flux data are available at the SAPFLUXNET global database (https://sapfluxnet.creaf.cat/) with codename ESP_MAJ.

1.6 Albedo-induced global warming potential ($GWP_{\Delta\alpha}$)

Assuming the $RF_{\Delta\alpha}$ at TOA is equal to longwave radiative forcing from CO_2, global warming potential of $\Delta\alpha_s$ ($GWP_{\Delta\alpha}$) was introduced to quantify the change in land surface due to nutrient manipulation into equivalent CO_2 emission or removal (Bright et al., 2015). Changes in albedo can be converted to CO_2-equivalents by integrating with time-horizon (Yu et al., 2024):

$$GWP_{\Delta\alpha} = \frac{S \cdot RF_{\Delta\alpha} \cdot C}{AF \cdot rf_{CO_2} \cdot TH} \quad (10)$$

where S is the perturbed area that is affected by albedo change. Here we assumed it as 1 ha, AF is the airborne fraction messing the increase of atmospheric CO_2 caused by anthropogenic CO2 emission, which equals to 0.5 in this analysis, rf_{CO2} is a grouped parameter with a constant value of 0.908 kg_{CO2}^{-1}. TH is time-horizon, which was set as 100 years as the IPCC standard. In this equation, $RF_{\Delta\alpha}$ (W m^{-2}) is local snapshot of surface change into TOA without translate into global mean (unit in 10^{-14} W m^{-2} m^{-2}).

1.7 LAI and nutrient concentration sampling

Every year from 2014 to 2016, the herbaceous layer was sampled in spring to measure biomass, leaf level nutrient concentration and LAI. The herbaceous biomass was harvested in multiple 25 x 25 cm squared within 25 x 25 m plots in each ecosystem footprints (see Fig 2 in El-Madany et al. (2021)). A subsample of the total biomass was selected and green and senescent fraction were separated, weighted, scanned, dried, and weighted again. Detailed calculation method for LAI can be found at El-Madany et al. (2021). Then, N concentration was analyzed by the dry combustion method (Vario EL: elementar GmbH, Hanau, Germany) using 25 mg of the ground sample material. P concentrations were analyzed using an inductive coupled plasma optical emission spectrometer (ICP OES, Optima 3300, Perkin Elmer). The leaf sample of the *Quercus ilex* trees were analyzed in the same way as he samples of herbaceous layer. The only difference were the sampling dates (Fig. S2). For the *Quercus ilex*, the leaf sampling was done during the winter period when the foliar nutrient concentrations are more stable. For nutrient analysis branches from the upper third of the crown (both North and South orientations) were detached and 100 leaves per tree were sampled. Between 6 and 8 trees were

sampled per site. Detailed laboratory procedure for nutrient concentration analysis can be found at El-Madany et al. (2021).

1.8 Soil respiration

In May 2015, 16 semi-automatic soil flux measurement chambers were installed in a stratified random sampling grouped into different nutrient addition treatments and canopy positions. We installed 8 chambers at NT and another 8 chambers for CT. At each site set 4 chambers located on open land and another 4 sets chambers below canopy. The chambers are in-house-developed stainless-steel design, connected to a LI-820 (LI-COR, Lincoln, Nebraska, USA), measuring in a half-hourly cycle. During this cycle one chamber at a time would close for a 3 min measurement duration. While there were 16 chambers in all, only data from CT and NT located at open land are displayed in this study. Fluxes and their variance were computed from CO_2 concentration time series using *ResChamberProc* R package. Additional details about the soil respiration processing can be found in Wutzler et al. (2020). The data also available at public data repository COSORE database under code nae ES-LMa (CT) and ES-LM1 (NT): https://github.com/bpbond/cosore (Bond-Lamberty et al., 2020)

2. Annual albedo-driven radiative forcing

Supplementary Table S1. The annual changes in surface albedo and the corresponding albedo-mediated radiative forcing ($RF_{\Delta\alpha}$ TOA and $GWP_{\Delta\alpha}$) due to nutrient additions (mean ± standard deviation). The albedo changes are the difference between the nutrient site and the control.

	Year	Albedo changes	Albedo-mediated radiative forcing		Albedo changes	Albedo-mediated radiative forcing	
			$RF_{\Delta\alpha}$ TOA (W m^{-2})	$GWP_{\Delta\alpha}$ (kg CO_2e ha^{-1} yr^{-1})		$RF_{\Delta\alpha}$ (W m^{-2})	$GWP_{\Delta\alpha}$ (kg CO_2e ha^{-1} yr^{-1})
		NT – CT			NPT – CT		
Ecosystem	2014	0.021 ± 0.004	-3.62 ± 1.11	-797.01	0.025 ± 0.003	-4.55 ± 1.64	-946.80
	2015	0.023 ± 0.007	-3.57 ± 1.48	-786.70	0.023 ± 0.009	-3.41 ± 1.05	-709.12
	2016	0.019 ± 0.009	-2.77 ± 0.94	-610.98	0.025 ± 0.011	-3.39 ± 0.81	-704.75
	2017	0.009 ± 0.011	-0.78 ± 1.58	-172.30	0.013 ± 0.014	-1.39 ± 1.46	-289.37
	2018	0.004 ± 0.006	-0.62 ± 1.09	-136.97	0.009 ± 0.005	-1.28 ± 0.78	-267.13
	2019	0.015 ± 0.005	-2.46 ± 1.15	-541.11	0.013 ± 0.007	-1.70 ± 0.59	-352.98
	2020	0.022 ± 0.009	-3.07 ± 0.96	-677.30	0.010 ± 0.015	-1.08 ± 2.34	-224.79
	2021	0.014 ± 0.011	-1.60 ± 1.50	-353.12	0.007 ± 0.013	-0.19 ± 2.16	-40.45
	2022	0.019 ± 0.008	-2.72 ± 1.08	-599.17	0.016 ± 0.009	-2.10 ± 0.76	-436.13
	2023	0.019 ± 0.008	-2.65 ± 0.64	-584.36	0.013 ± 0.014	-1.40 ± 1.14	-291.22
		mean	-2.39 ± 1.05	-525.90 ± 232.58		-2.05 ± 1.33	-426.27 ± 276.03
Understory	2015	0.038 ± 0.003	-2.97 ± 0.64	-617.01	0.041± 0.001	-3.21 ± 0.555	-666.62
	2016	0.016 ± 0.009	-2.35 ± 1.60	-487.80	0.015 ± 0.016	-1.52 ± 1.75	-316.21
	2017	0.013 ± 0.006	-1.93 ± 1.08	-401.42	0.017 ± 0.007	-2.49 ± 1.03	-518.19
	2018	0.016 ± 0.006	-2.59 ± 1.41	-539.31	0.010 ± 0.005	-1.61 ± 0.87	-335.63
	2019	0.017 ± 0.004	-2.71 ± 1.18	-563.09	0.011 ± 0.005	-1.82 ± 0.96	-378.87
	2020	0.018 ± 0.005	-3.02 ± 1.31	-627.36	0.011 ± 0.006	-2.07 ± 1.30	-430.04
	2021	0.018 ± 0.005	-3.03 ± 1.71	-630.17	0.016 ± 0.008	-2.27 ± 0.80	-473.21
	2022	0.022 ± 0.006	-3.94 ± 1.89	-818.01	0.029 ± 0.005	-5.22 ± 2.96	-1086.35
	2023	0.015 ± 0.006	-2.66 ± 1.66	-554.08	0.017 ± 0.010	-2.26 ± 1.15	-470.57
		mean	-2.80 ± 0.55	-582.03 ± 114.97		-2.50 ± 1.14	-519.52 ± 237.28

3. DTW temporal similarity analysis

Supplementary Table S2. DTW distance analysis of eco-physiology components on surface temperature change (mean ± SD) at different fertilizations

				autumn	*winter*	*spring*	*drydown*	*summer*	*autumn*	*winter*	*spring*	*drydown*	*summer*
		Ecosystem	ΔTs (°C)	-0.07 ± 0.27	-0.07 ± 0.19	-0.52 ± 0.44	-1.02 ± 0.41	-0.64 ± 0.28	0.25 ± 0.27	0.07 ± 0.22	-0.16 ± 0.23	-0.20 ± 0.24	0.03 ± 0.21
				δε (0.02)	δε (0.03)	δG (0.03)	δα (0.02)	δα (0.03)	δε (0.02)	δε (0.04)	δε (0.02)	δε (0.01)	δε (0.02)
				δα (0.05)	δG (0.08)	δε (0.03)	δG (0.05)	δG (0.05)	δLE (0.06)	δG (0.07)	δG (0.02)	δα (0.02)	δG (0.03)
				δLE (0.06)	δLE (0.10)	δα (0.03)	δLE (0.06)	δε (0.05)	δG (0.07)	δLE (0.08)	δα (0.03)	δG (0.02)	δα (0.05)
				δG (0.07)	δH (0.12)	δH (0.06)	δε (0.07)	δLE (0.07)	δH (0.07)	δH (0.10)	δH (0.05)	δLE (0.05)	δLE (0.08)
				δH (0.08)	δα (0.12)	δLE (0.06)	δH (0.07)	δH (0.14)	δI (0.08)	δI (0.13)	δLE (0.06)	δH (0.06)	δH (0.15)
				δI (0.12)	δI (0.16)	δI (0.11)	δI (0.09)	δI (0.20)	δα (0.09)	δα (0.18)	δI (0.09)	δI (0.09)	δI (0.17)
ΔTs	NT – CT	Grass	ΔTs (°C)	0.35 ± 0.28	0.39 ± 0.24	0.59 ± 0.41	0.90 ± 0.51	0.93 ± 0.40	0.38 ± 0.41	0.14 ± 0.26	0.56 ± 0.56	1.40 ± 0.75	1.52 ± 0.52
				δε (0.10)	δε (0.14)	δG (0.09)	δG (0.15)	δG (0.19)	δε (0.09)	δε (0.10)	δG (0.08)	δG (0.26)	δG (0.29)
				δSWDR (0.15)	δSWDR (0.15)	δε (0.12)	δε (0.28)	δε (0.41)	δG (0.14)	δG (0.12)	δLWDR (0.11)	δLWDR (0.37)	δLE (0.49)
				δG (0.18)	δG (0.20)	δLWDR (0.19)	δLWDR (0.35)	δLWDR (0.43)	δLWDR (0.17)	δα (0.18)	δε (0.11)	δε (0.40)	δSWDR (0.50)
				δLWDR (0.26)	δLWDR (0.26)	δα (0.19)	δα (0.42)	δLE (0.59)	δα (0.19)	δLWDR (0.20)	δα (0.18)	δα (0.40)	δε (0.58)
				δα (0.27)	δα (0.37)	δH (0.27)	δH (0.42)	δH (0.68)	δSWDR (0.21)	δSWDR (0.24)	δH (0.27)	δH (0.52)	δLWDR (0.76)
				δLE (0.31)	δH (0.48)	δLE (0.49)	δLE (0.59)	δα (0.98)	δLE (0.27)	δH (0.32)	δSWDR (0.40)	δLE (0.53)	δα (0.82)
				δH (0.35)	δLE (0.53)	δSWDR (0.57)	δSWDR (0.89)	δSWDR (1.06)	δH (0.28)	δLE (0.45)	δI (0.51)	δSWDR (0.62)	δH (0.94)
				δI (0.58)	δI (0.81)	δI (0.68)	δI (1.64)	δI (1.72)	δI (0.55)	δI (0.98)	δLE (0.63)	δI (0.86)	δI (0.99)

A lower value means the component is closer to ΔTs; it represents that the component has a similar pattern and is closely related. See Supplementary Table 1 for a more complete DTW value for all eco-physiology components.

4. Measurement setup and pre-nutrient addition status

4.1 Soil nutrient characteristic before nutrient addition

Supplementary Table S3. Top soil chemical characterization (0 – 10 cm depth) in the region of Extremadura (mean ± std). This data originated from Schnabel et al. (2013).

Soil property	***Unit***	***Mean ± stdev***
pH		5.43 ± 0.46
Cation Exchange Capacity (CEC)	(cmol kg^{-1})	8.3 ± 3.3
Calium (Ca)	(cmol kg^{-1})	3.3 ± 2.4
Magnesium (Mg)	(cmol kg^{-1})	1.0 ± 1.1
Potassium (K)	(cmol kg^{-1})	0.2 ± 0.2
Sodium (Na)	(cmol kg^{-1})	0.7 ± 0.4
Base saturation	(%)	66.5 ± 35.8
Nitrogen (N)	(g kg^{-1})	1.0 ± 0.6
Phosphorus (P)	(g kg^{-1})	5.8 ± 9.4
Soil Organic Carbon (SOC)	(g kg^{-1})	11.6 ± 4.6

4.2 Majadas measurement set up

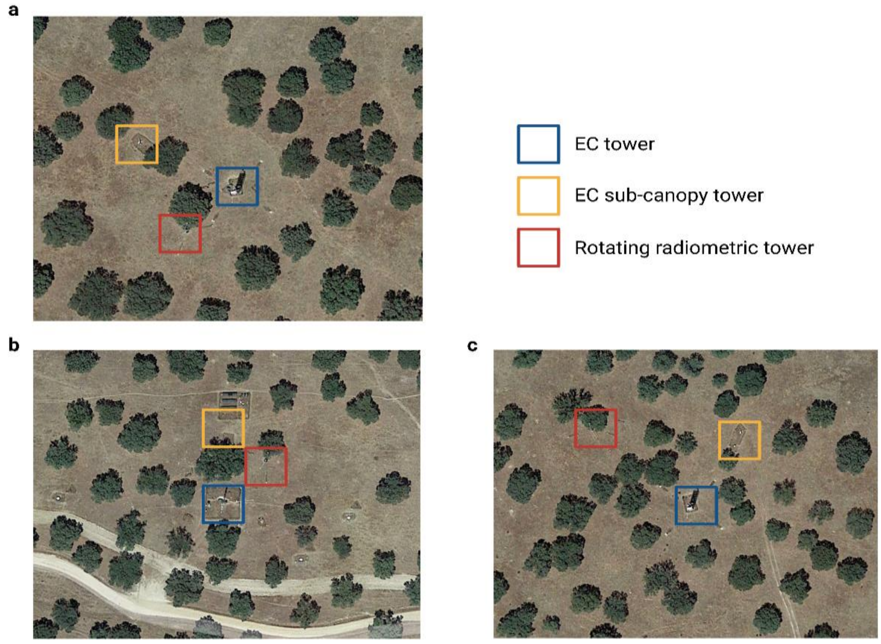

Supplementary Fig S1. Majadas de Tietar measurement set up for a) NT, b) CT, and c) NPT. Each site consisted of a high-tower with EC system at 15 m height (blue square), sub-canopy EC system at 1.5 m height for the grass layer (yellow square), and a rotating radiometric tower to monitor net radiation components above the grass and tree layer (red square).

4.3 Radiometric tower set up

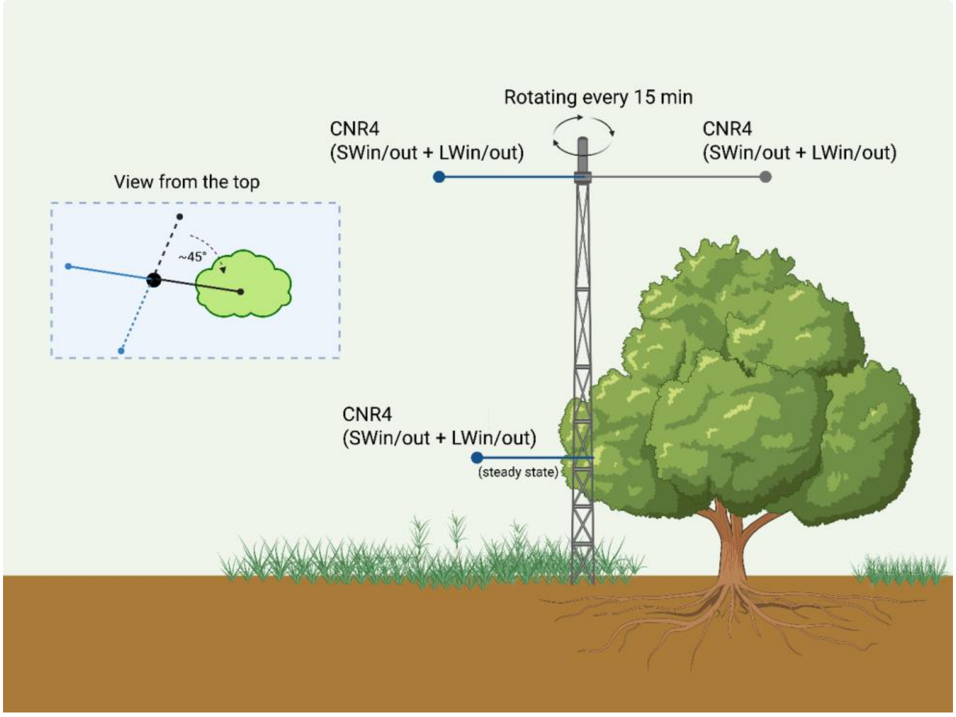

Supplementary Fig S2. Illustration of rotating radiometric tower to measure incoming and outgoing radiation over a tree top and above a grass layer. CNR4 is a four-component net radiometer. SW is shortwave radiation and LW is longwave radiation.

4.4 Near infrared index for trees before and after nutrient addition

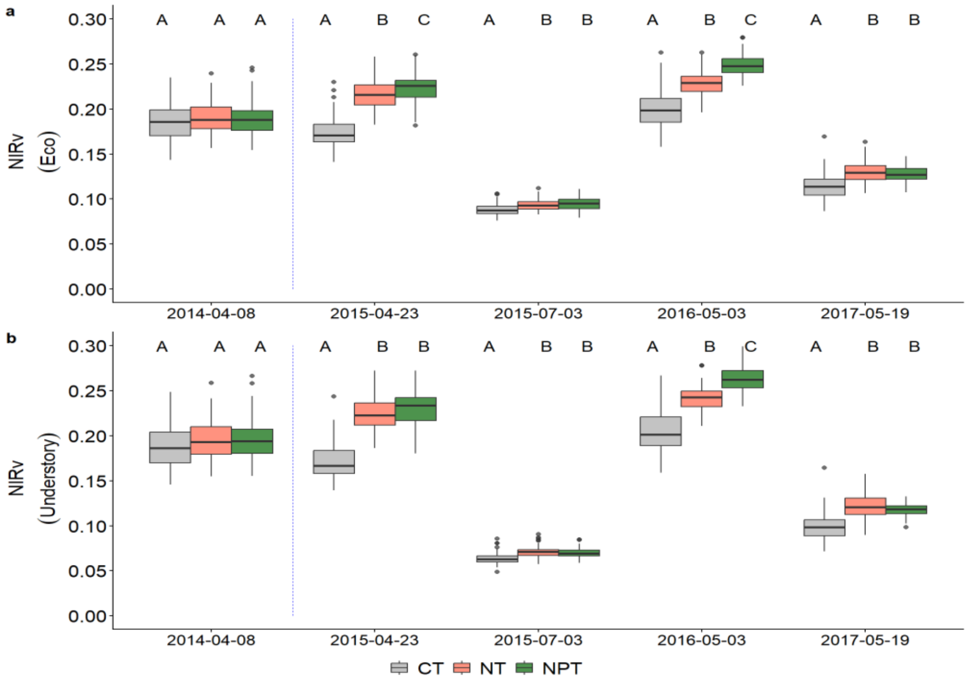

Supplementary Fig S3. Ecosystem and understory NIRv based on airborne data analysis. The vertical blue dashed line represents the start of the nutrient addition experiment. Letters on the boxplot represent significant differences between sites.

4.5 Ecosystem NEE correlation between sites before nutrient addition

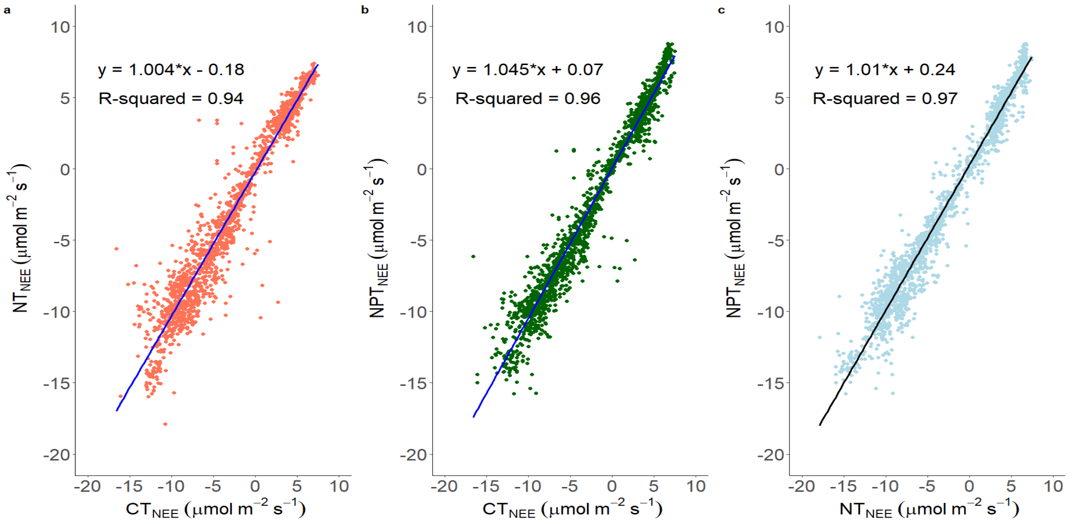

Supplementary Fig S4. Correlation of half-hourly NEE (μmol $m^{-2}s^{-1}$) between CT and NT (a), CT and NPT (b), NT and NPT (c) for spring 2014 (peak growing period) before nutrient addition treatment started.

4.6 Ecosystem albedo correlation between sites before nutrient addition for midday albedo uncertainty

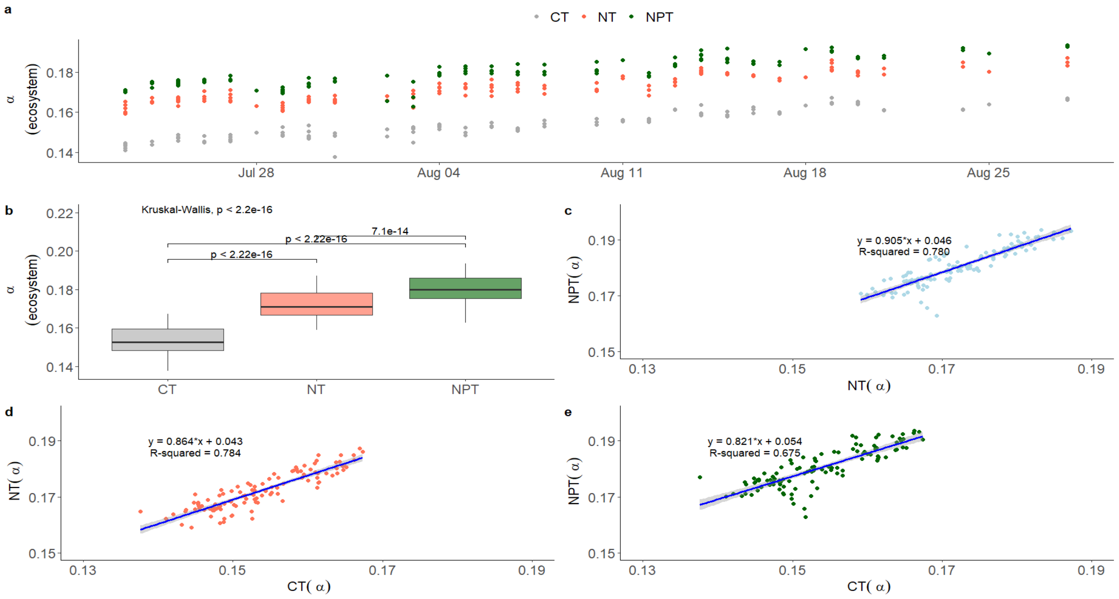

Supplementary Fig S5. Timeseries half-hourly midday albedo (11:00 – 14:30) (a), comparison of midday albedo between tower (b) and correlation between towers (NT-NPT (c), CT-NT (d), CT-NPT (e)) during summer 2014 (assuming grass layer fully senescent during this period).

5. Site terrain and windrose

5.1 Digital elevation model map

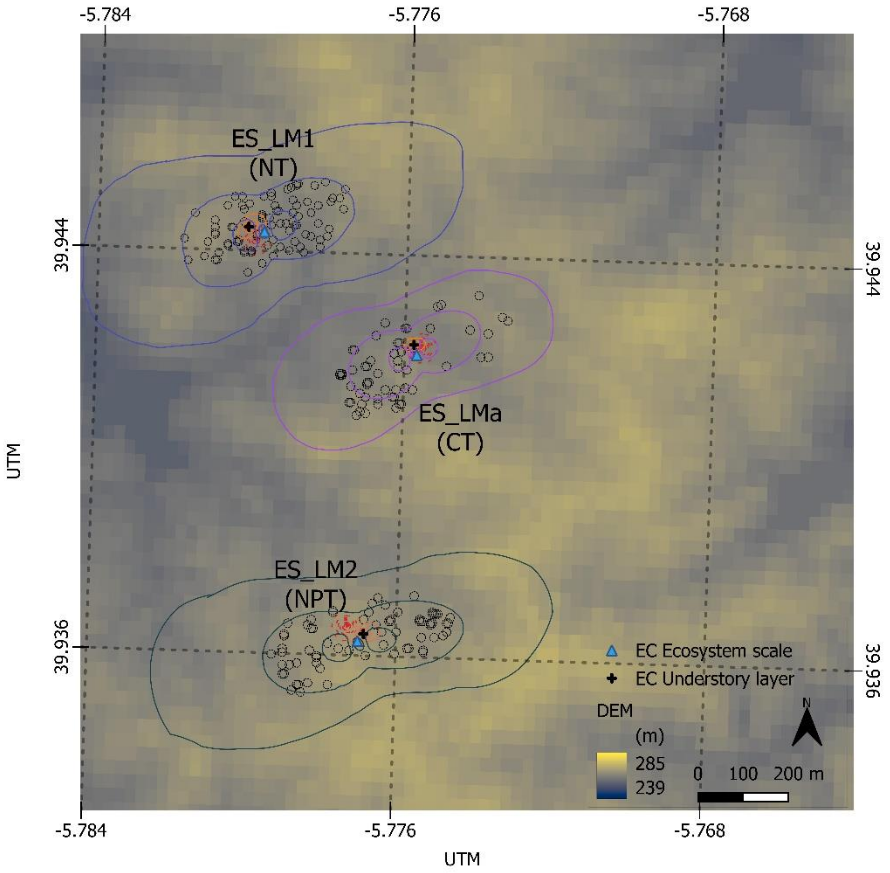

Supplementary Fig S6. Terrain map based on the digital elevation model (DEM) at Majadas de Tietar. Blue triangle represents ecosystem EC tower, while red cross pointed out the sub-canopy EC. The circle represents selected tree points to calculate tree-grass vegetation index (Fig S9 and S10) pre and after-nutrient addition treatment

5.2 Windrose for each EC tower

a)

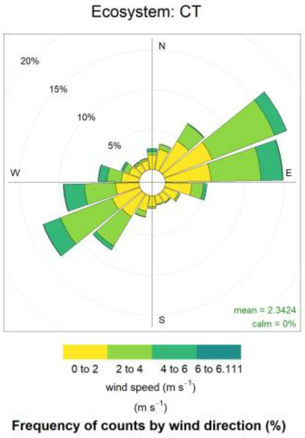

b)

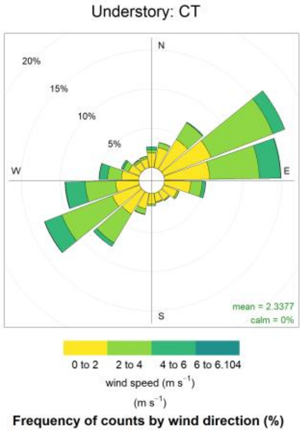

c)

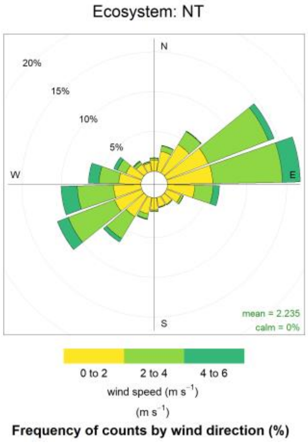

d)

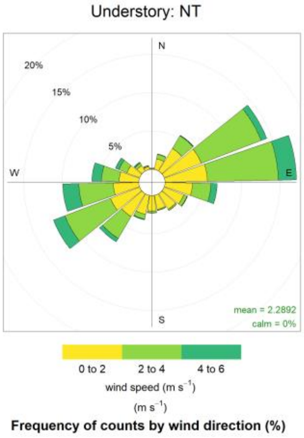

e)

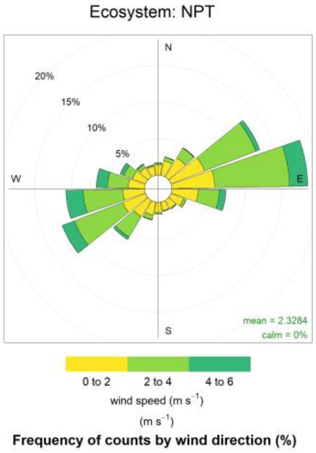

f)

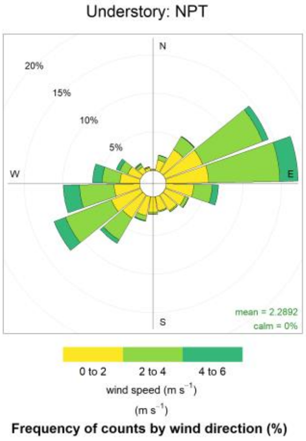

Supplementary Fig S7. Ecosystem and understory wind roses for CT (a, b), NT (c, d), and NPT (e, f), respectively

6. Timeline of nutrient addition

Supplementary Table S4. Timeline and amount (kg ha^{-1}) of nutrient addition at Majadas de Tiétar.

Date	2014	2015				2016				2017			
	Autumn	Winter	Spring	Summer	Autumn	Winter	Spring	Summer	Autumn	Winter	Spring	Summer	Autumn
N		100				20				50			
P	50				10				25				

Date	2018				2019				2020			
	Winter	Spring	Summer	Autumn	Winter	Spring	Summer	Autumn	Winter	Spring	Summer	Autumn
N												
P								6				6

Date	2021				2022				2023			
	Winter	Spring	Summer	Autumn	Winter	Spring	Summer	Autumn	Winter	Spring	Summer	Autumn
N	24								12			
P								6				

7. Leaf nutrient content and LAI

7.1 Herbaceous LAI

Supplementary Table S5. Herbaceous leaf area index (LAI, mean ± std) and midday α across the sites (CT-control, NT-nitrogen only addition, and NPT-nitrogen and phosphorus addition).

Sampling date	LAI (m^2 m^{-2})			Midday α						season
				Ecosystem			Understory			
	CT	NT	NPT	CT	NT	NPT	CT	NT	NPT	
08-04-2014	1.54 ± 0.46	1.46 ± 0.45	1.35 ± 0.56	0.125	0.146	0.147				spring
23-04-2015	1.54 ± 0.41	1.66 ± 0.61	2.14 ± 0.60	0.142	0.162	0.163				spring
08-07-2015	0.13 ± 0.13	0.18 ± 0.13	0.29 ± 0.17	0.152	0.171	0.172				summer
03-05-2016	1.91 ± 0.63	3.24 ± 1.68	2.74 ± 1.46	0.138	0.152	0.159	0.177	0.182	0.178	spring
20-05-2016	2.64 ± 1.04	2.58 ± 2.81	1.87 ± 0.21	0.133	0.144	0.145	0.264	0.174	0.164	drydown
18-05-2017	0.89 ± 0.37	1.07 ± 0.21	1.16 ± 0.19	0.128	0.137	0.132	0.155	0.159	0.156	drydown
17-07-2018	0.16 ± 0.12	0.20 ± 0.17	0.24 ± 0.16	0.154	0.148	0.154	0.175	0.190	0.187	summer

7.2 Herbaceous layer nutrient concentration

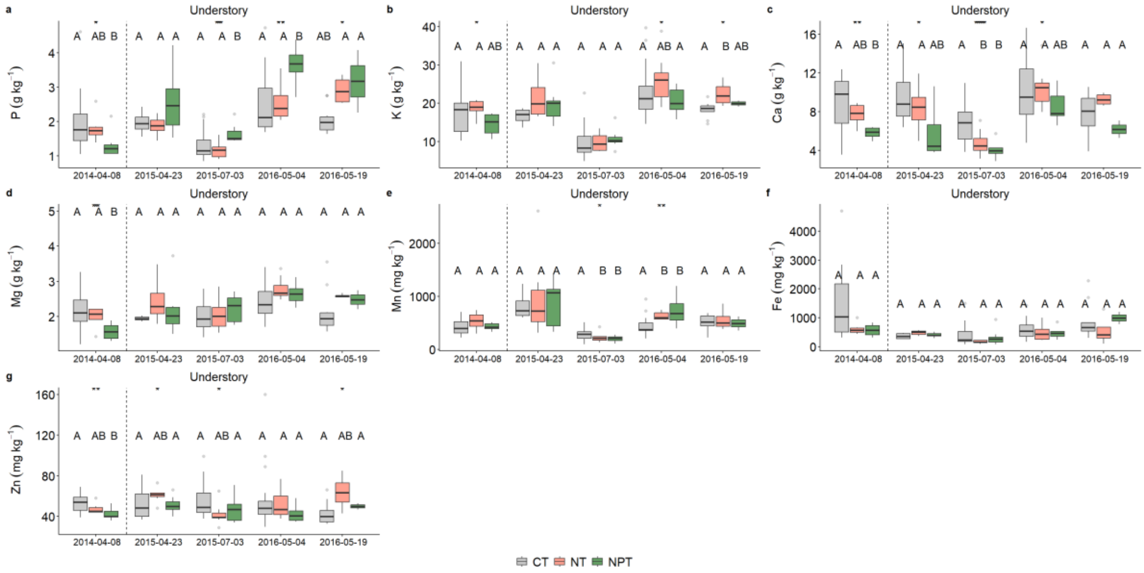

Supplementary Fig S8. Herbaceous phosphorous (P), potassium (K), calcium (Ca), magnesium (Mg), manganese (Mn), iron (Fe), and zinc (Zn) concentrations on the specific sampling dates of the herbaceous layer at each site. Letters above the boxplot represent the significant difference ($p < 0.05$). The vertical dotted line symbolizes the start of nutrient addition.

7.3 Tree leaf nutrient concentration

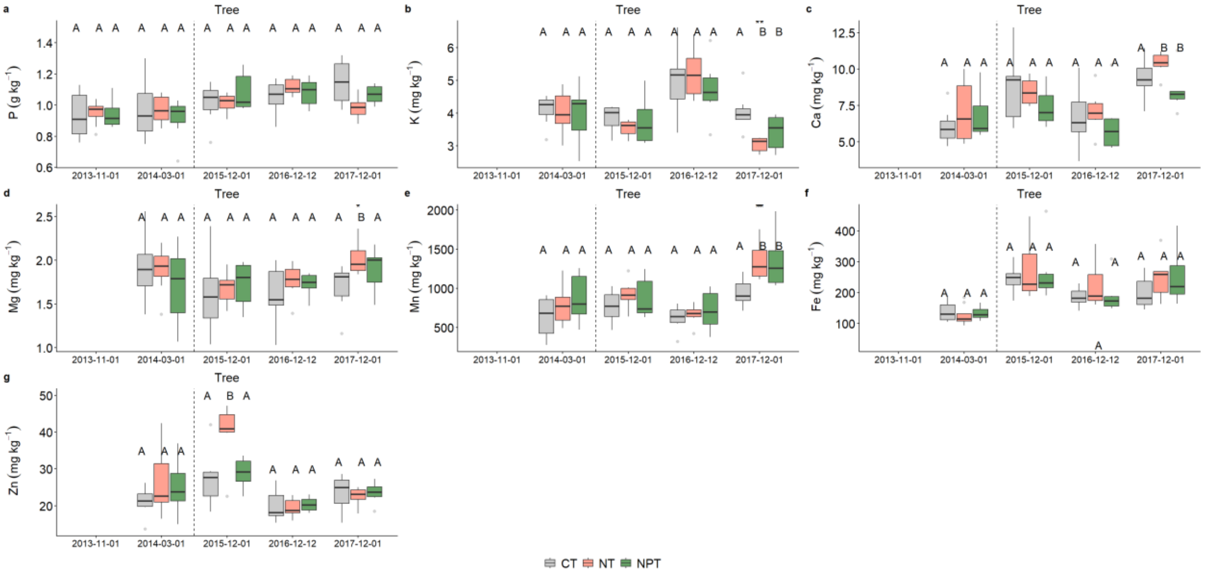

Supplementary Fig S9. Tree leaf phosphorous (P), potassium (K), calcium (Ca), magnesium (Mg), manganese (Mn), iron (Fe), and zinc (Zn) concentrations on the specific sampling dates of the tree leaf at each site. Letters above the boxplot represent the significant difference ($p < 0.05$). The vertical dotted line symbolizes the start of nutrient addition.

7.4 Leaf N, C and C:N ratio

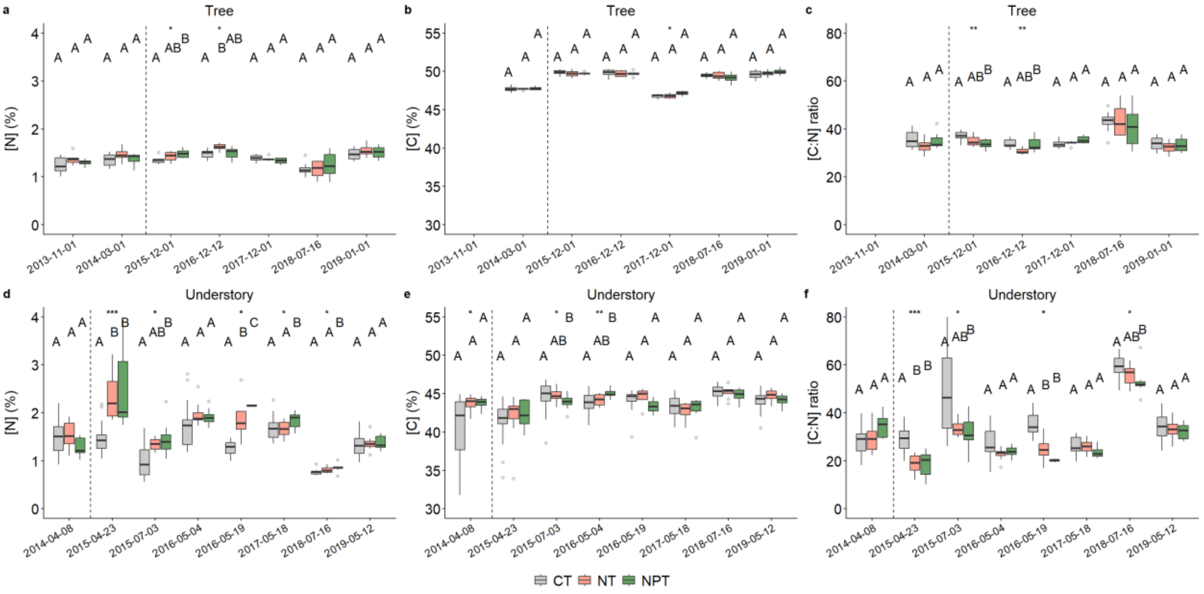

Supplementary Fig S10. Leaf nitrogen (N) concentration, leaf carbon (C) concentration, and leaf C:N ratio on the specific sampling dates of tree (a, b, c) and understory (d, e, f) at each site. Letters above the boxplot represent the significant difference ($p < 0.05$). The vertical dotted line symbolizes the start of nutrient addition.

7.5 Leaf N and P correlation with NEE and albedo

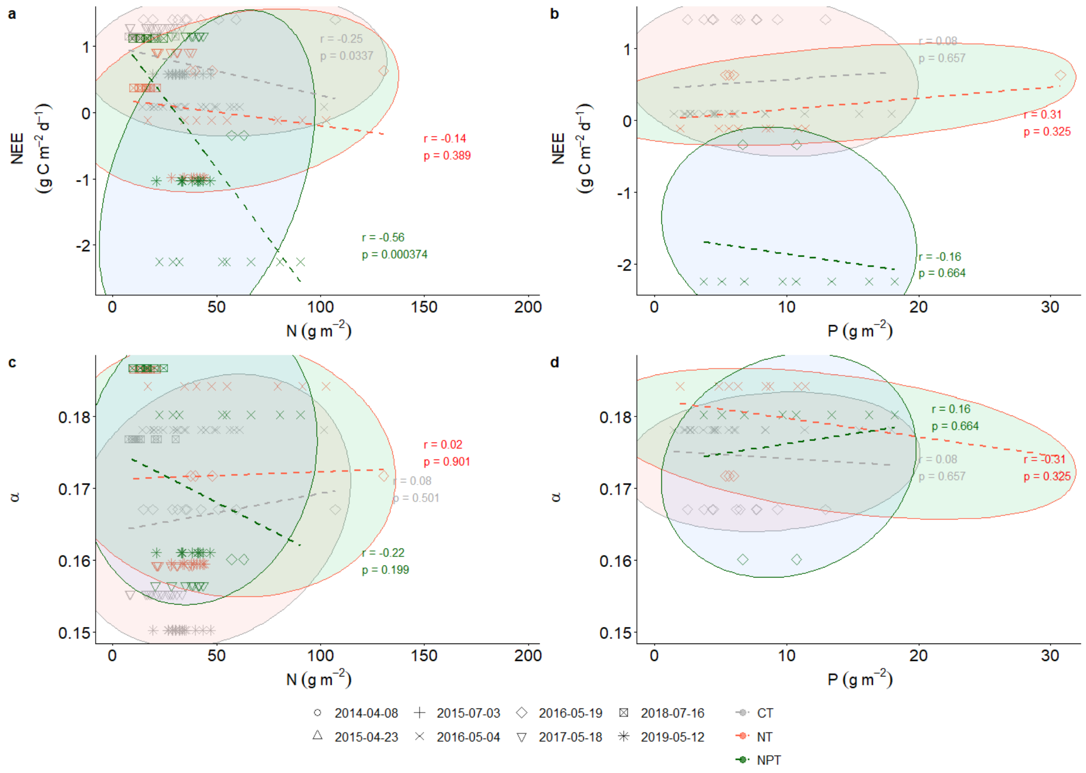

Supplementary Fig S11. Correlation analysis between herbaceous leaf nitrogen (N) and phosphorus (P) concentrations with sub-canopy net ecosystem exchange (NEE) and grass albedo (α) of field collected samples. The r values represent the correlation coefficient and p values mean significant level of correlation coefficient.

8. Time series of soil respiration

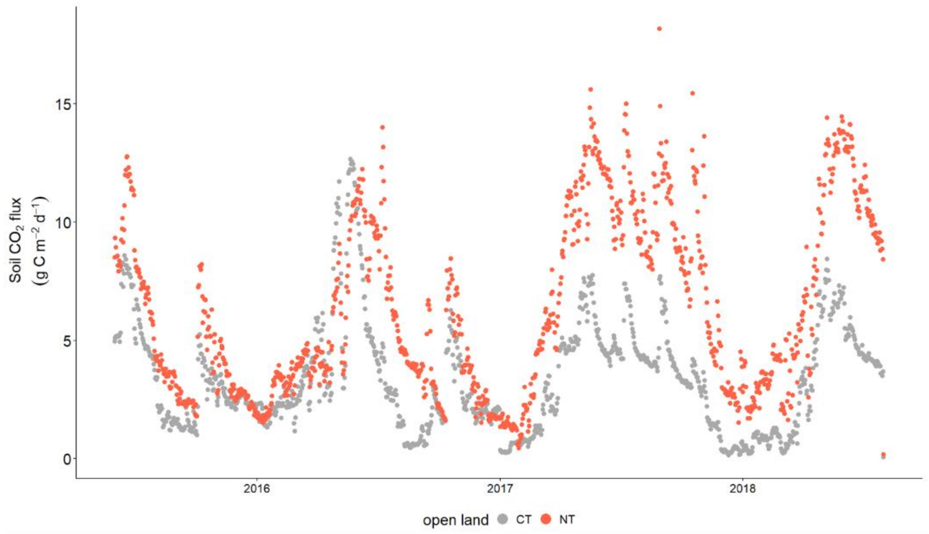

Supplementary Fig S12. Daily soil respiration (i.e., soil CO_2 flux) between the control (CT) and N-only addition (NT) sites from 2016 to 2018.

9. Percentage of gap-filled NEE, quality flag and daily partitioning

9.1 Percentage of gap-filled

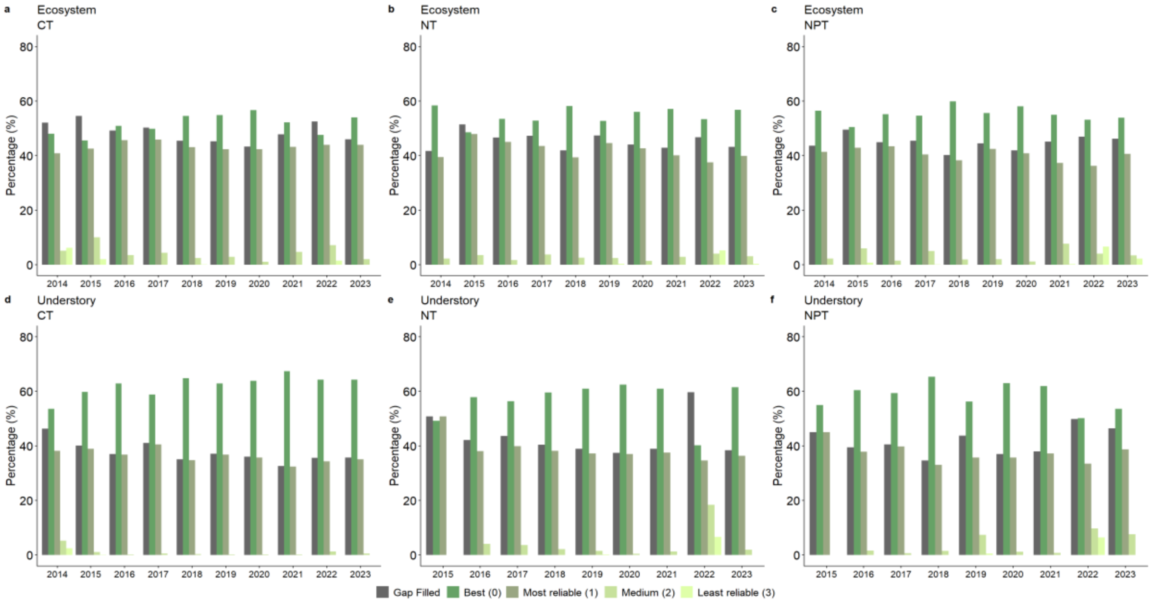

Supplementary Fig S13. Annual percentage of gap-filled NEE and quality flag for each tower.

9.2 Time series daily GPP

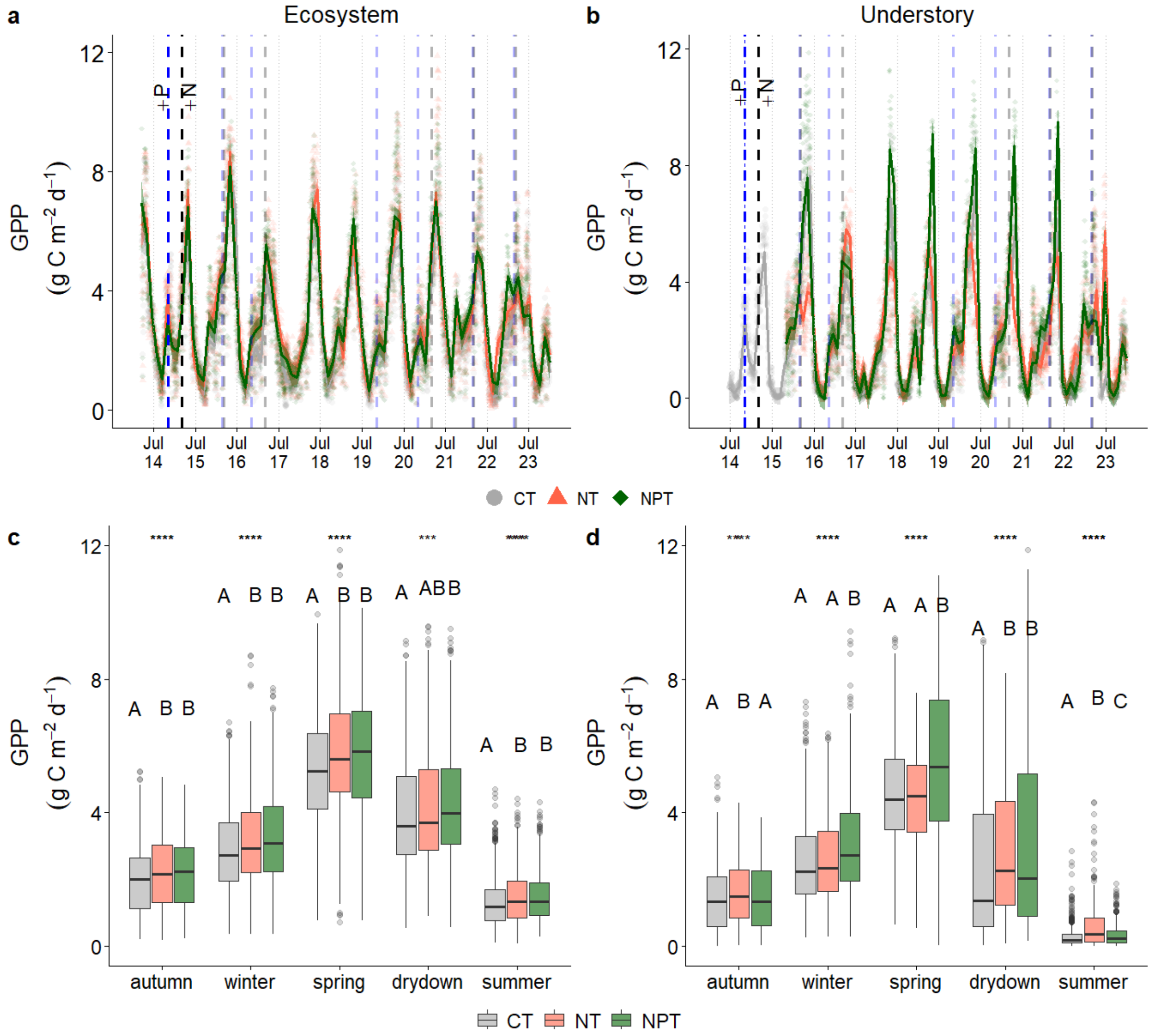

Supplementary Fig S14. Time series daily GPP under different nutrient addition at ecosystem-scale (a), grass layer (b), and respective season (c, d) at ecosystem and grass layer. The vertical blue dashed line represents the date nutrient experiment. Letters on the boxplot represent significant differences between sites.

9.3 Time series Reco

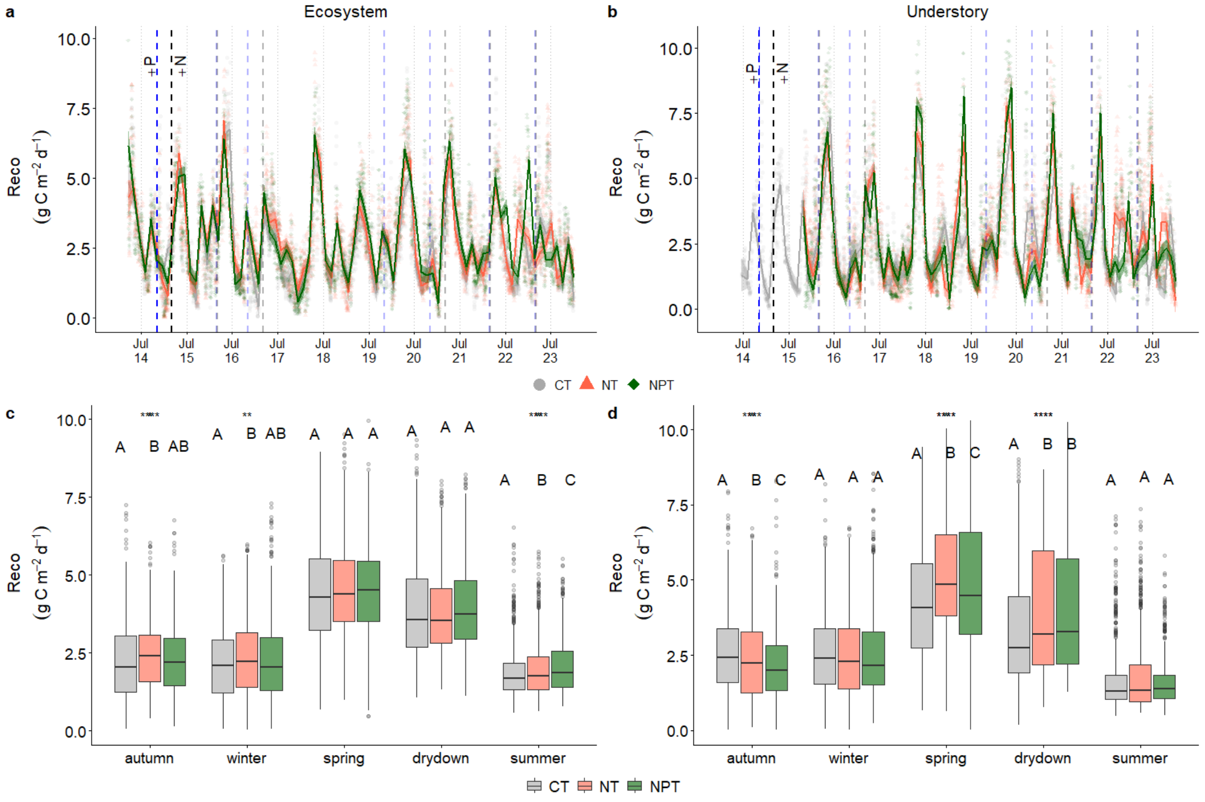

Supplementary Fig S15. Time series daily Reco under different nutrient addition at ecosystem-scale (a), grass layer (b), and respective season (c, d) at ecosystem and grass layer. The vertical blue dashed line represents the date nutrient experiment. Letters on the boxplot represent significant differences between sites

10. Seasonal characteristics of Majadas based on phenocam

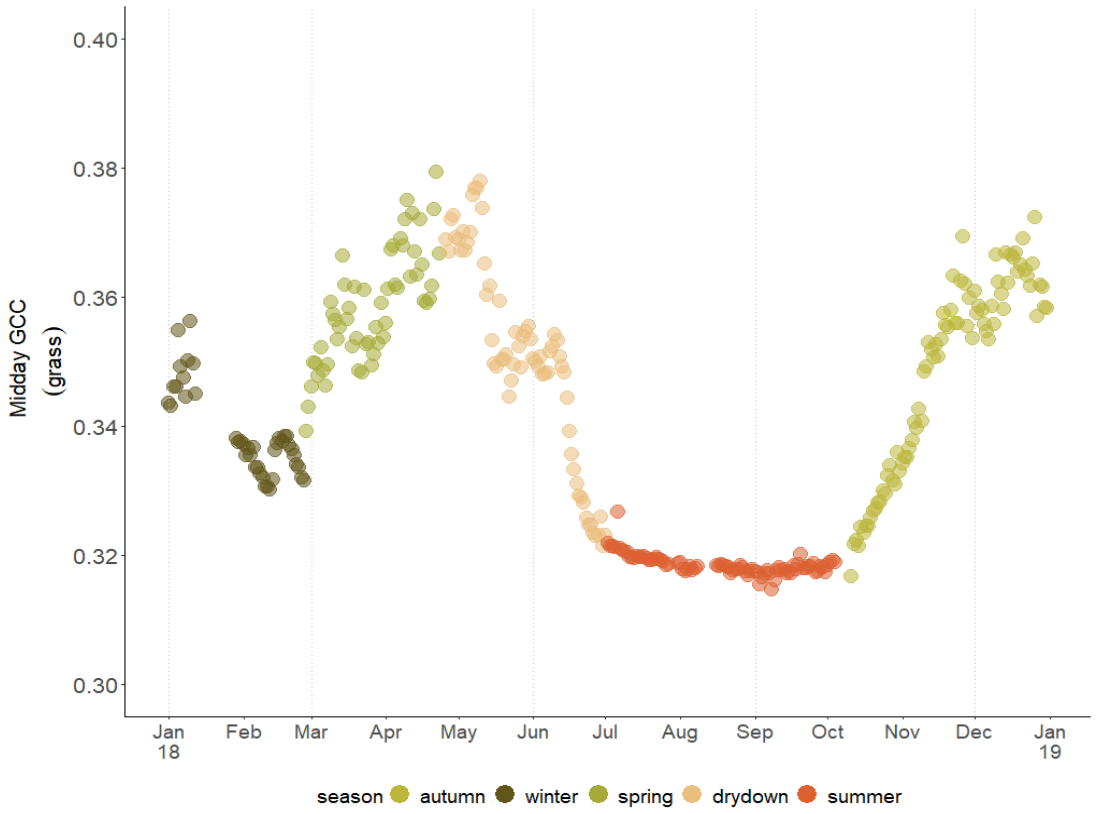

Supplementary Fig S16. The typical time series of daily midday GCC (green chromatic coordinates) from the grass layer as indicator for phenological seasons.

11. Radiative kernel comparison with surface SWDR

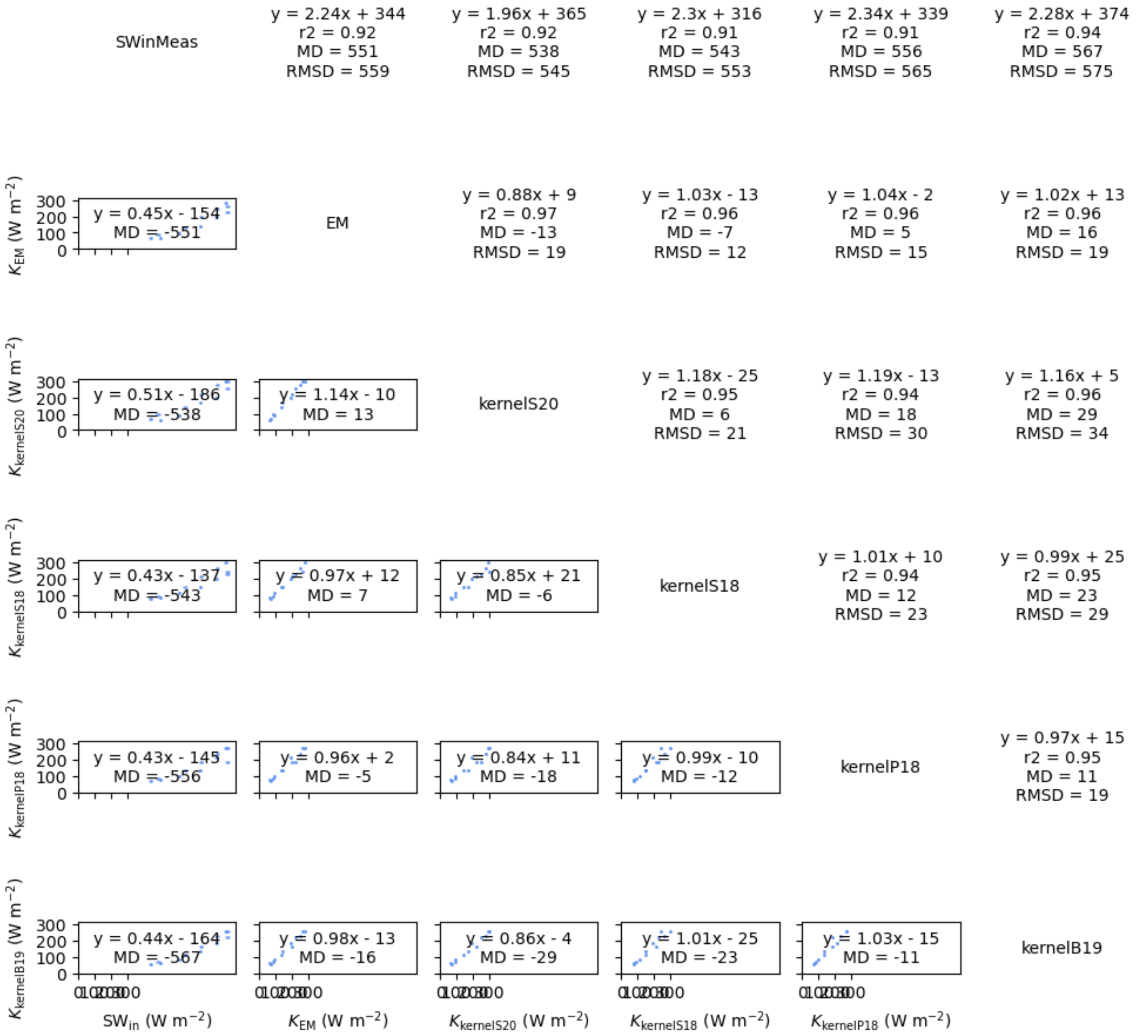

Supplementary Fig S17. Comparison between measured SWDR at the surface (CT), surface albedo kernels from four different datasets at Majadas de Tietar and their ensemble mean. KernelS20 represents HadGEM3, kernelS18 is HadGEM2, kernelP18 is CAM5, and kernelB19 contains CACK v1.0 radiative kernel. Each data point (blue-dots) corresponds to a monthly climatology data, r2: coefficient of determination (squared Pearson correlation coefficient), MD: mean difference, RMSD: Root mean square difference.

12. Comparison between ΔTs observed from CRN4 sensor and ΔTs calculated

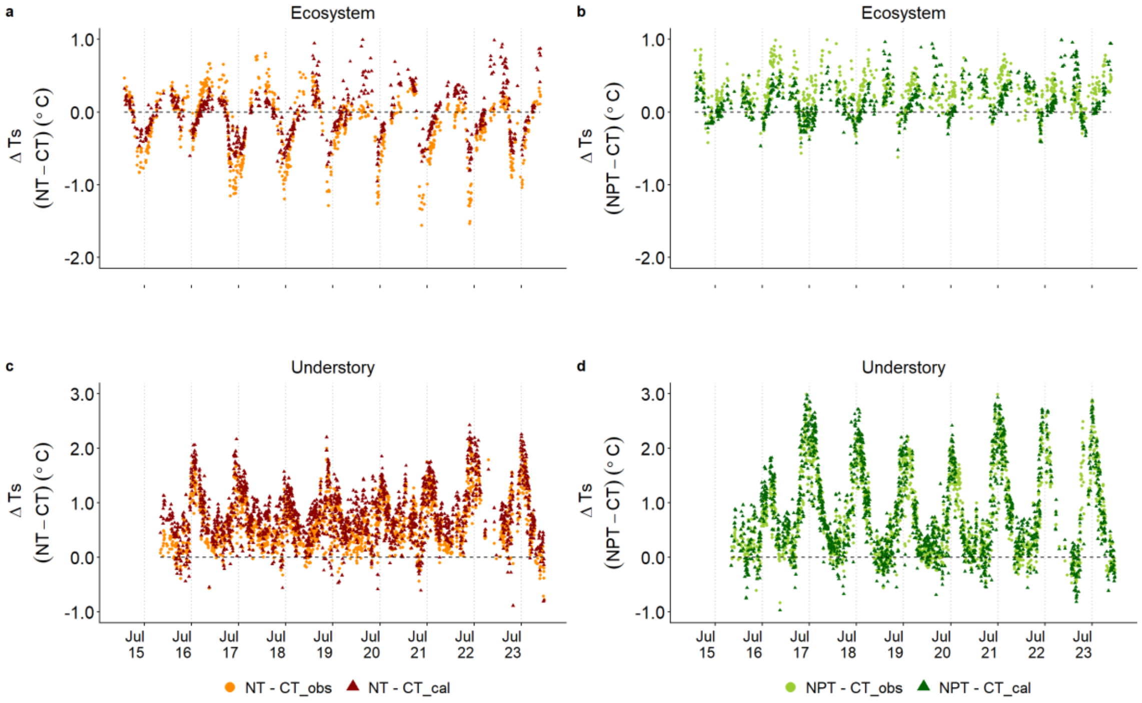

Supplementary Fig S18. Comparison between ΔTs observation from CNR4 sensor and ΔTs calculation based on equation 10. It compares the fertilization site to the control site on each ecosystem and grass layer. The vertical blue dashed line represents the start of the nutrient addition experiment.

13. Evaporative fraction and evapotranspiration

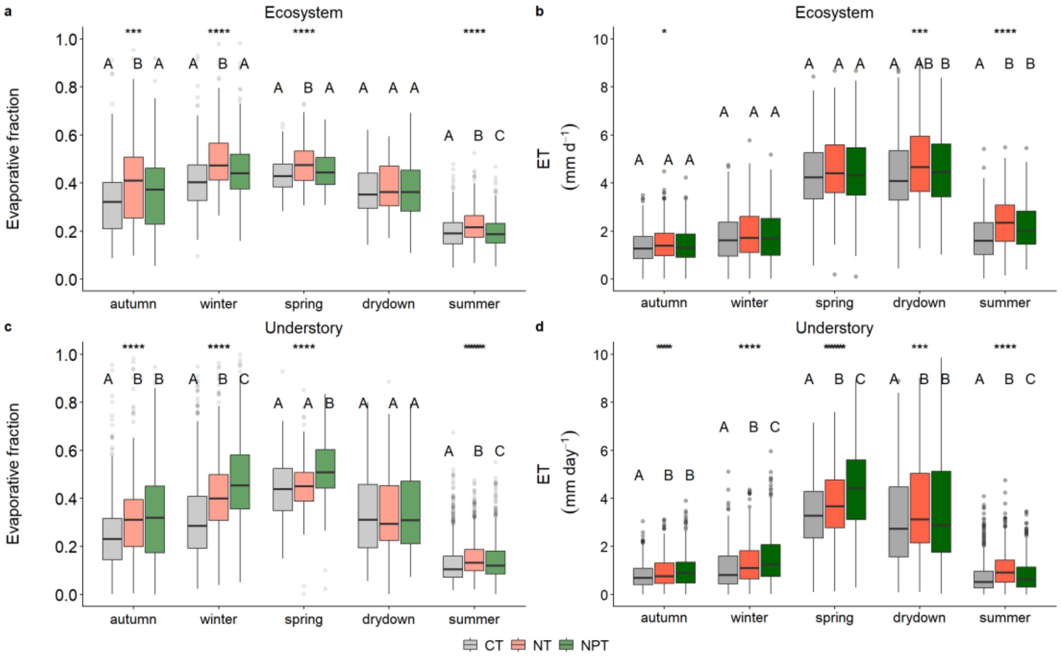

Supplementary Fig S19. Daily evaporation fraction and evapotranspiration (ET) between towers on the ecosystem scale and grass layer. The vertical blue dashed line represents the start of the nutrient addition experiment. Letters on the boxplot represent significant differences between sites.

14. Energy balance closure

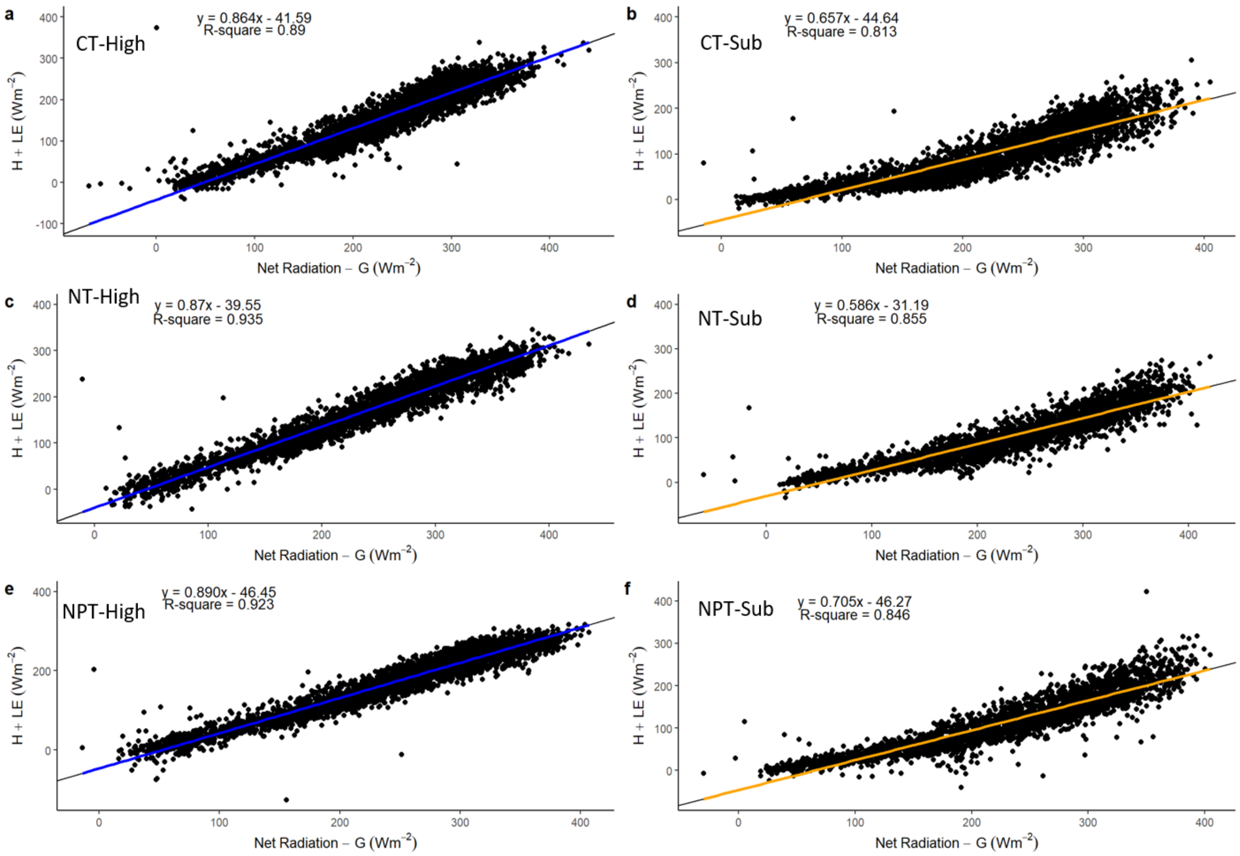

Supplementary Fig S20. Half-hourly energy balance closure between towers on ecosystem (high) and understory layer (sub-canopy).

15. Tree layer response on nutrient addition

15.1 Daily midday α and ΔTs on tree

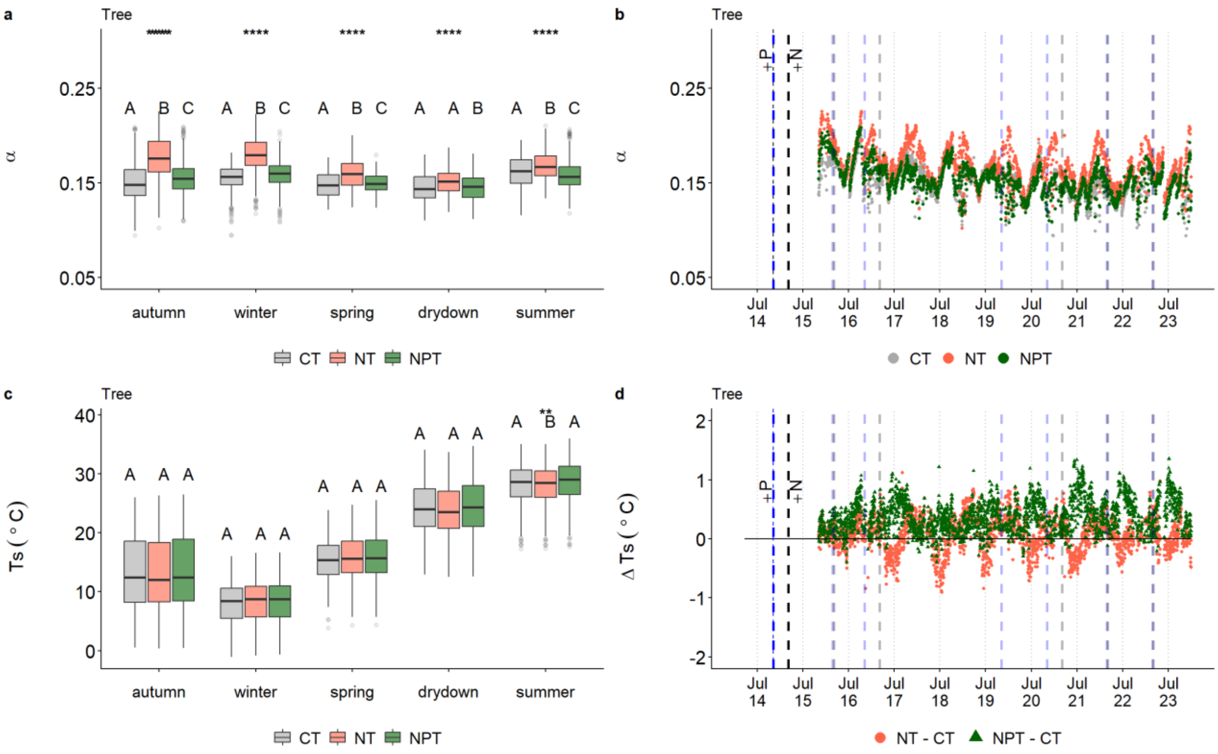

Supplementary Fig S21. Daily midday albedo and daily surface temperature (Ts) between towers on tree layer (radiometric tower). The vertical blue dashed line represents the start of the nutrient addition experiment. Letters on the boxplot represent significant differences between sites

15.2 Comparison ΔTs observation and ΔTs calculation on tree

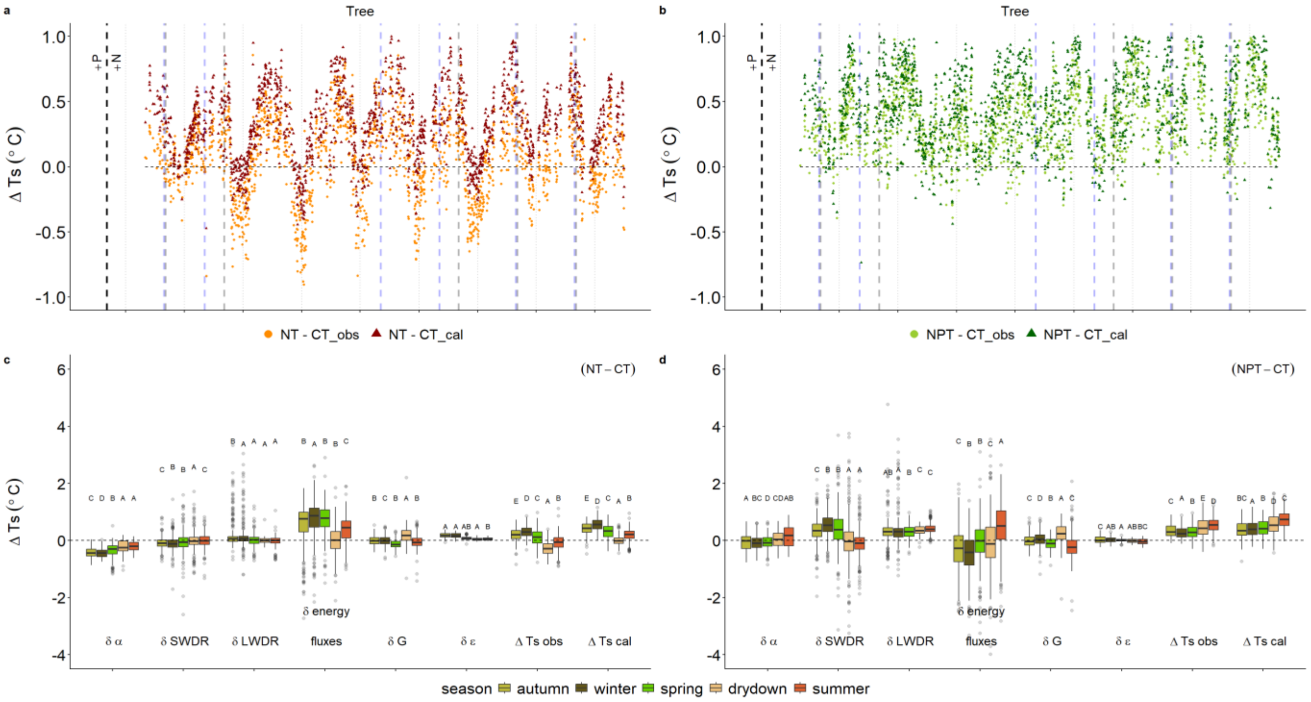

Supplementary Fig S22. Comparison ΔTs observation and ΔTs calculation on tree between fertilization site and control site on tree layer (a,b); eco-physiology components on surface temperature change on tree layer. The vertical blue dashed line represents the start of the nutrient addition experiment. Letters on the boxplot represent significant differences between sites.

15.3 Radiative forcing on tree

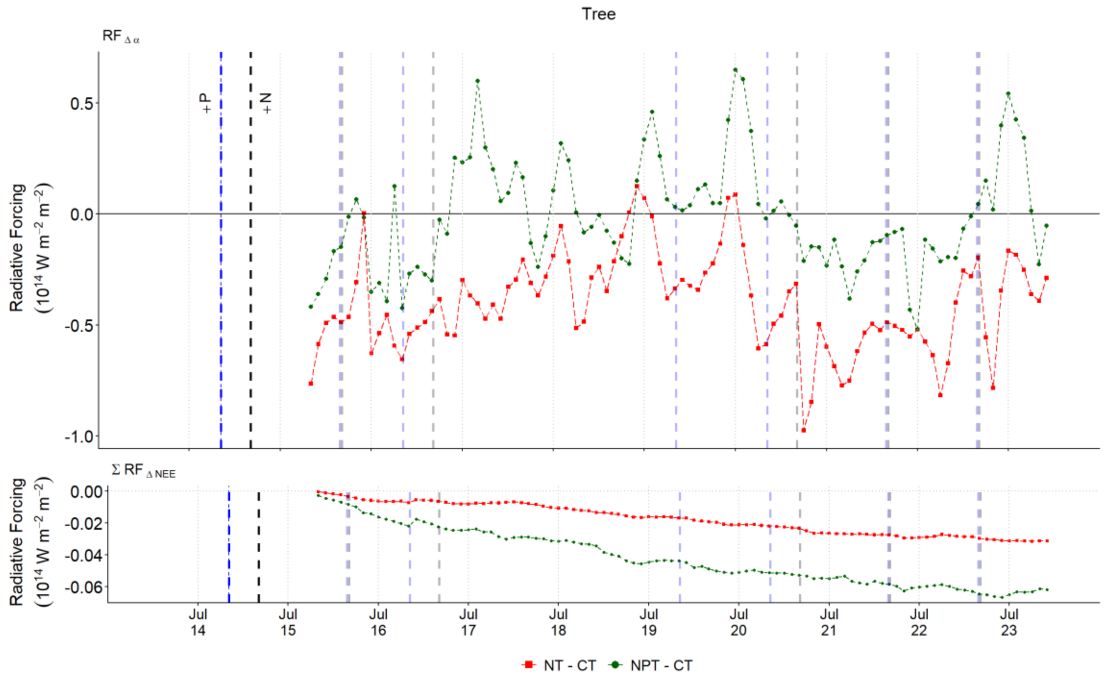

Supplementary Fig S23. Radiative forcing due to albedo and NEE changes on tree layer corresponding to fertilization treatments. The vertical blue dashed line represents the start of the nutrient addition experiment.

15.4 Tree sapflux transpiration

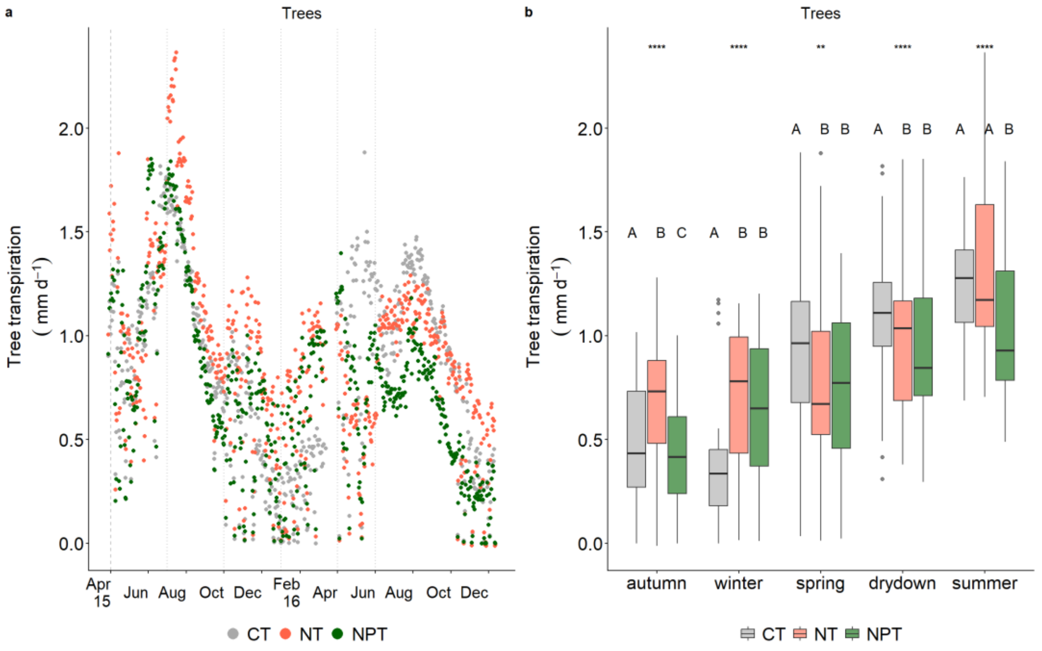

Supplementary Fig S24. Daily sapflux at the three towers (CT-control, NT-nitrogen only addition, and NPT-nitrogen and phosphorus addition) from spring 2015 to 2016. Letters on the boxplot represent significant differences between sites (post-hoc HSD, $p < 0.05$). Dots in the boxplot represent outlier, horizontal lines inside the boxes are the median values, and the box borders show the interquartile ranges of the data.

16. Summer albedo anomaly

16.1 Understory α during summer

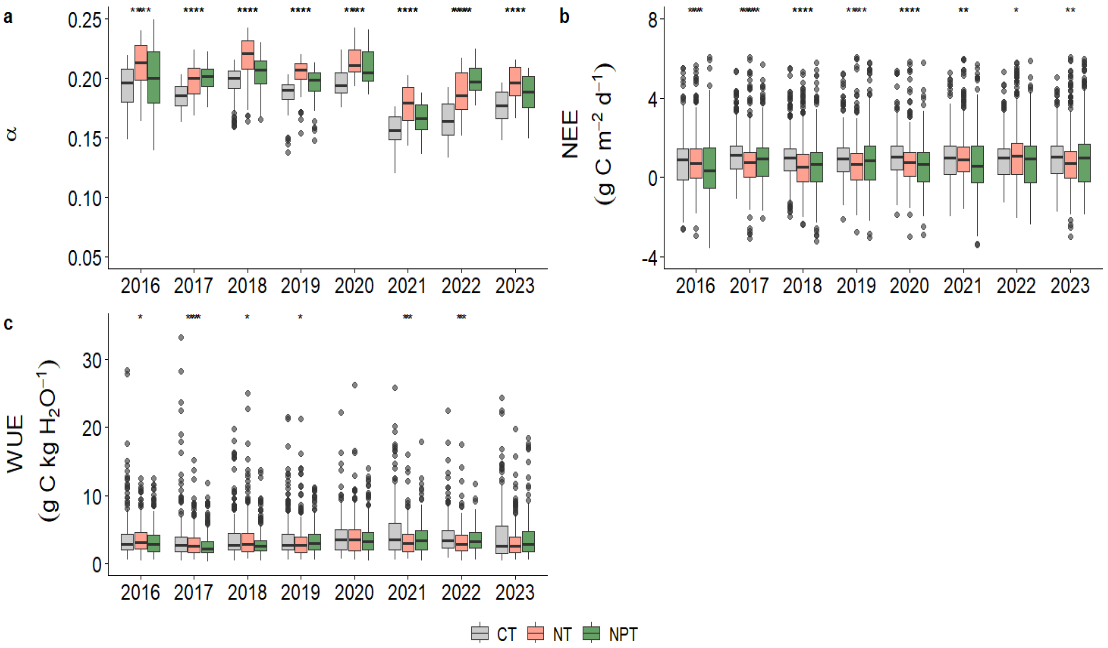

Supplementary Fig S25. Time series midday α on understory during summer periods from 2016 to 2023. Dots in the boxplot represent outlier, horizontal lines inside the boxes are the median values, and the box borders show the interquartile ranges of the data.

17. References

Badgley, G., Field, C. and Berry, J.A., 2017. Canopy near-infrared reflectance and terrestrial photosynthesis. Science Advances, 3: 1-5.

Bond-Lamberty, B. et al., 2020. COSORE: A community database for continuous soil respiration and other soil-atmosphere greenhouse gas flux data. Glob Chang Biol, 26(12): 7268-7283.

Bright, R.M., Zhao, K., Jackson, R.B. and Cherubini, F., 2015. Quantifying surface albedo and other direct biogeophysical climate forcings of forestry activities. Glob Chang Biol, 21(9): 3246-66.

Burchard-Levine, V. et al., 2021. The effect of pixel heterogeneity for remote sensing based retrievals of evapotranspiration in a semi-arid tree-grass ecosystem. Remote Sensing of Environment, 260.

El-Madany, T.S. et al., 2018. Drivers of spatio-temporal variability of carbon dioxide and energy fluxes in a Mediterranean savanna ecosystem. Agricultural and Forest Meteorology, 262: 258-278.

El-Madany, T.S. et al., 2021. How Nitrogen and Phosphorus Availability Change Water Use Efficiency in a Mediterranean Savanna Ecosystem. Journal of Geophysical Research: Biogeosciences, 126(5).

Foken, T., 2008. The energy balance closure problem: an overview. Ecol Appl, 18(6): 1351-67.

Gentine, P., Entekhabi, D., Chehbouni, A., Boulet, G. and Duchemin, B., 2007. Analysis of evaporative fraction diurnal behaviour. Agricultural and Forest Meteorology, 143(1-2): 13-29.

Juang, J.Y., Katul, G., Siqueira, M., Stoy, P. and Novick, K., 2007. Separating the effects of albedo from eco-physiological changes on surface temperature along a successional chronosequence in the southeastern United States. Geophysical Research Letters, 34(21).

Luyssaert, S. et al., 2014. Land management and land-cover change have impacts of similar magnitude on surface temperature. Nature Climate Change, 4(5): 389-393.

Nutini, F., Boschetti, M., Candiani, G., Bocchi, S. and Brivio, P., 2014. Evaporative Fraction as an Indicator of Moisture Condition and Water Stress Status in Semi-Arid Rangeland Ecosystems. Remote Sensing, 6(7): 6300-6323.

Pacheco-Labrador, J. et al., 2017. Spatio-Temporal Relationships between Optical Information and Carbon Fluxes in a Mediterranean Tree-Grass Ecosystem. Remote Sensing, 9(6).

Perez-Priego, O. et al., 2017. Evaluation of eddy covariance latent heat fluxes with independent lysimeter and sapflow estimates in a Mediterranean savannah ecosystem. Agricultural and Forest Meteorology, 236: 87-99.

Rouse Jr, J., Haas, R.H., Schell, J.A. and Deering, D.W., 1974. Monitoring vegetation systems in the great plains with ERTS.

Schnabel, S., Dahlgren, R.A. and Moreno, G., 2013. Soil and water dynamics. In: P. Campos et al. (Editors), Mediterranean Oak Woodland Working Landscapes. Dehesas of Spain and Ranchlands of California. Springer, pp. 91 - 121.

Tong, B. et al., 2022. Effects of soil moisture, net radiation, and atmospheric vapor pressure deficit on surface evaporation fraction at a semi-arid grass site. Sci Total Environ, 849: 157890.

Weiser, U., Olefs, M., Schöner, W., Weyss, G. and Hynek, B., 2016. Correction of broadband snow albedo measurements affected by unknown slope and sensor tilts. The Cryosphere, 10(2): 775-790.

Wood, J.D., Griffis, T.J. and Baker, J.M., 2015. Detecting drift bias and exposure errors in solar and photosynthetically active radiation data. Agricultural and Forest Meteorology, 206: 33-44.

Wutzler, T., Perez-Priego, O., Morris, K., El-Madany, T.S. and Migliavacca, M., 2020. Soil CO2 efflux errors are lognormally distributed – implications and guidance. Geoscientific Instrumentation, Methods and Data Systems, 9(1): 239-254.

Yu, K. et al., 2024. Quantifying albedo impact and radiative forcing of management practices in European wheat cropping systems. Environmental Research Letters, 19(7).

Zhang, Q. et al., 2020. Reforestation and surface cooling in temperate zones: Mechanisms and implications. Global Change Biology, 26(6): 3384-3401.
